# Combining single-cell RNA sequencing and population-based studies reveals hand osteoarthritis-associated chondrocyte subpopulations and pathways

**DOI:** 10.1038/s41413-023-00292-7

**Published:** 2023-11-02

**Authors:** Hui Li, Xiaofeng Jiang, Yongbing Xiao, Yuqing Zhang, Weiya Zhang, Michael Doherty, Jacquelyn Nestor, Changjun Li, Jing Ye, Tingting Sha, Houchen Lyu, Jie Wei, Chao Zeng, Guanghua Lei

**Affiliations:** 1 Department of Orthopaedics, Xiangya Hospital, Central South University, Changsha, 410008 Hunan China; 2 Hunan Key Laboratory of Joint Degeneration and Injury, Changsha, 410008 Hunan China; 3 Key Laboratory of Aging-related Bone and Joint Diseases Prevention and Treatment, Ministry of Education, Xiangya Hospital, Central South University, Changsha, China; 4 Division of Rheumatology, Allergy, and Immunology, Department of Medicine, Massachusetts General Hospital, Harvard Medical School, Boston, MA 02115 USA; 5 The Mongan Institute, Massachusetts General Hospital, Harvard Medical School, Boston, MA 02115 USA; 6 Academic Rheumatology, School of Medicine, University of Nottingham, Nottingham, NG5 1PB UK; 7 Pain Centre Versus Arthritis UK, Nottingham, NG5 1PB UK; 8 Department of Endocrinology, Endocrinology Research Center, Xiangya Hospital, Central South University, Changsha, 410008 Hunan China; 9 National Clinical Research Center for Geriatric Disorders, Xiangya Hospital, Central South University, Changsha, 410008 Hunan China; 10 Health Management Center, Xiangya Hospital, Central South University, Changsha, 410008 Hunan China; 11 Department of Epidemiology and Health Statistics, Xiangya School of Public Health, Central South University, Changsha, 410008 Hunan China

**Keywords:** Homeostasis, Pathogenesis

## Abstract

Hand osteoarthritis is a common heterogeneous joint disorder with unclear molecular mechanisms and no disease-modifying drugs. In this study, we performed single-cell RNA sequencing analysis to compare the cellular composition and subpopulation-specific gene expression between cartilage with macroscopically confirmed osteoarthritis (*n* = 5) and cartilage without osteoarthritis (*n* = 5) from the interphalangeal joints of five donors. Of 105 142 cells, we identified 13 subpopulations, including a novel subpopulation with inflammation-modulating potential annotated as inflammatory chondrocytes. Fibrocartilage chondrocytes exhibited extensive alteration of gene expression patterns in osteoarthritic cartilage compared with nonosteoarthritic cartilage. Both inflammatory chondrocytes and fibrocartilage chondrocytes showed a trend toward increased numbers in osteoarthritic cartilage. In these two subpopulations from osteoarthritic cartilage, the ferroptosis pathway was enriched, and expression of iron overload-related genes, e.g., *FTH1*, was elevated. To verify these findings, we conducted a Mendelian randomization study using UK Biobank and a population-based cross-sectional study using data collected from Xiangya Osteoarthritis Study. Genetic predisposition toward higher expression of *FTH1* mRNA significantly increased the risk of hand osteoarthritis (odds ratio = 1.07, 95% confidence interval: 1.02–1.11) among participants (*n* = 332 668) in UK Biobank. High levels of serum ferritin (encoded by *FTH1*), a biomarker of body iron overload, were significantly associated with a high prevalence of hand osteoarthritis among participants (*n* = 1 241) of Xiangya Osteoarthritis Study (*P*-for-trend = 0.037). In conclusion, our findings indicate that inflammatory and fibrocartilage chondrocytes are key subpopulations and that ferroptosis may be a key pathway in hand osteoarthritis, providing new insights into the pathophysiology and potential therapeutic targets of hand osteoarthritis.

## Introduction

Hand osteoarthritis (OA) is a common heterogeneous joint disorder and involves a distinctive OA phenotype with differences in etiology and pathophysiology from knee OA and hip OA.^1–5^ Patients with hand OA frequently report symptoms of pain, functional limitations and disability in daily activities.^6,7^ Previous studies have shown that the clinical burden of hand OA is comparable to that of rheumatoid arthritis.^8,9^ During the past few years, several clinical trials^10–13^ have been conducted to assess the efficacy of symptomatic slow-acting and anti-inflammatory drugs on hand OA, but the results are disappointing.^14,15^ To date, there is no known cure for hand OA, indicating a need for a better understanding of its underlying mechanisms such that appropriate prevention and treatment strategies can be developed to target this common form of arthritis.^2,14,15^

Single-cell RNA sequencing (scRNA-seq) provides an opportunity for comprehensive and unbiased characterization of cellular and molecular profiles in both healthy and diseased tissues.^16^ Identifying underlying disease-dependent differences at single-cell resolution can greatly help in understanding the molecular mechanisms and discovering target cells and pathways. Population-based studies provide evidence that is generalizable, increasing the robustness of scRNA-seq analysis findings. Moreover, a Mendelian randomization (MR) study is a technique that uses genetic variables as proxies for the exposure of interest, which are randomly allocated at conception and conditional on parental genotypes, and thus is able to estimate the causative effect of an exposure variable on an outcome while minimizing the risk of confounding and reverse causation.^17^ The combination of target discovery by scRNA-seq with validation by large population-based studies may help in translating research from the molecular to population level and ultimately in guiding development of effective prevention and treatment strategies for hand OA.

In this study, we performed scRNA-seq analysis on cartilage collected from macroscopically osteoarthritic and nonosteoarthritic interphalangeal joints from the hands of five human donors to compare alterations in cellular composition and subpopulation-specific gene expression. We further conducted (1) a MR study using data from UK Biobank to investigate the causal association between key differentially expressed gene and hand OA and (2) a cross-sectional study using data collected from a community-based observational study (Xiangya Osteoarthritis Study^18,19^) to examine the association between a serum biomarker (encoded by a key gene) and hand OA.

## Results

### Cell clusters in human hand articular cartilage

**Fig. 1:**
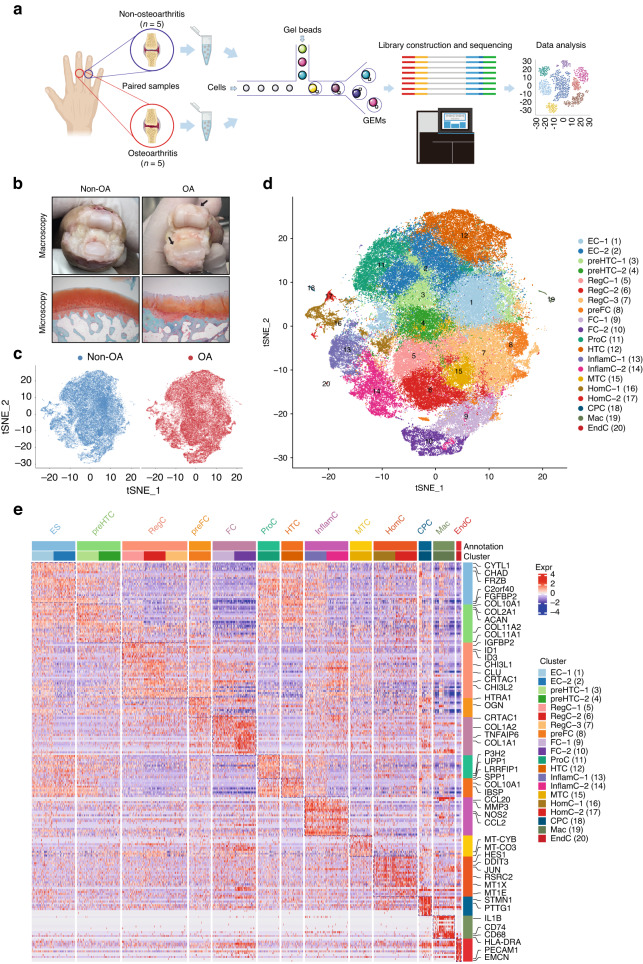
ScRNA-seq atlas of cell clusters in human hand articular cartilage. **a** Schematic workflow of single-cell RNA sequencing. **b** Representative macroscopic and microscopy images of hand osteoarthritic and nonosteoarthritic cartilage of hand interphalangeal joints. Arrows indicate locations of cartilage degeneration. **c** t-SNE embedding plot of cells colored according to disease status. All clusters contained cells from both hand osteoarthritic and nonosteoarthritic samples. **d** t-SNE embedding plot for 105 142 cells derived from paired hand articular cartilage samples of five donors. **e** Heatmap showing the top 10 discriminative genes of each cell cluster in hand articular cartilage. Genes indicated in the right column were adopted for annotation for each subpopulation. ScRNA-seq single-cell RNA sequencing, EC effector chondrocytes, preHTC prehypertrophic chondrocytes, RegC regulatory chondrocytes, preFC prefibrocartilage chondrocytes, FC fibrocartilage chondrocytes, HTC hypertrophic chondrocytes, InflamC inflammatory chondrocytes, MTC mitochondrial chondrocytes, HomC homeostatic chondrocytes, CPC cartilage progenitor cells, Mac macrophages, EndC endothelial cells

**Fig. 2:**
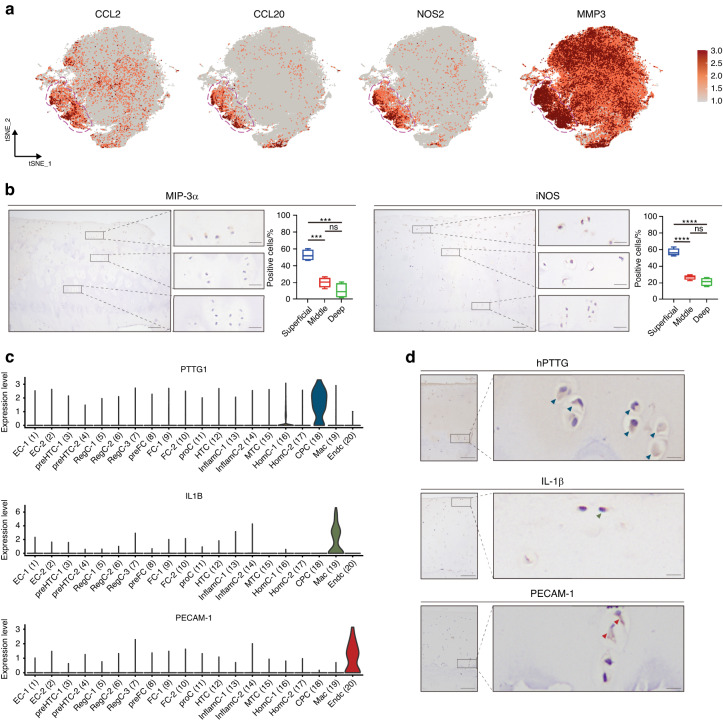
Signatures and putative spatial distribution of InflamC, CPC, Mac and EndC. **a** Expression of selected marker genes (combined for annotation) for InflamC visualized by a feature plot. **b** Representative immunohistochemistry (IHC) staining for MIP-3α and iNOS in hand articular cartilage tissues (*n* = 4). Scale bar, left, 100 μm; right, 20 μm. ***P < 0.001, ****P < 0.000 1. **c** Violin plot of selected marker genes for CPC, Mac and EndC. **d** Representative IHC staining of hPTTG, IL-1β and PECAM-1. Arrows indicate positive cells in the cartilage tissue. Scale bar, left, 100 μm; right, 10 μm. InflamC inflammatory chondrocytes, CPC cartilage progenitor cells, Mac macrophages, EndC endothelial cells, MIP-3α macrophage inflammatory protein 3 alpha, iNOS inducible nitric oxide synthase, hPTTG human pituitary tumor-transforming gene 1 protein, IL-1β interleukin-1 beta, PECAM-1 platelet endothelial cell adhesion molecule

We obtained 10 articular cartilage specimens (i.e., five osteoarthritic and five nonosteoarthritic specimens) from the interphalangeal joints of five donors (Fig. 1a and Table S1) whose pathological conditions were confirmed by macroscopic observation and Safranin-O/Fast Green staining (Fig. 1b). Among them, 105 142 individual cells passed the strict quality filtering process for subsequent analysis (Fig. S1), with 52 197 cells originating from osteoarthritic cartilage and 52 945 from nonosteoarthritic cartilage (Fig. 1c). We identified 20 putative clusters, including 18 chondrocyte clusters and two rare clusters, using the unsupervised method (Fig. 1d). The cluster-specific differentially expressed genes (DEGs) are shown in Table S2. Most chondrocyte clusters align with the following 10 chondrocyte subpopulations according to established markers (Fig. 1e)^20–24^: effector chondrocytes (EC, Cluster 1 and 2); prehypertrophic chondrocytes (preHTC, Cluster 3 and 4); regulatory chondrocytes (RegC, Cluster 5, 6, and 7); prefibrocartilage chondrocytes (preFC, Cluster 8); fibrocartilage chondrocytes (FC, Cluster 9, 10, and 11); proliferating chondrocytes (ProC, Cluster 12); hypertrophic chondrocytes (HTC, Cluster 13); mitochondrial chondrocytes (MTC, Cluster 16); homeostatic chondrocytes (HomC, Cluster 17 and 18); and cartilage progenitor cells (CPC, Cluster 19). Remaining Clusters 13 and 14 were grouped as a novel subpopulation (inflammatory chondrocytes, InflamC) according to their distinct expression of genes related to the inflammatory response, immune system process and immune response, i.e., *CCL20*, *CCL2*, *NOS2*, and *MMP3* (Fig. 2a). Immunohistochemistry (IHC) analysis revealed a predisposed distribution of InflamC in the superficial zone of articular cartilage (Fig. 2b). In addition, two small subpopulations were detected, including a subpopulation of macrophages (Mac, Cluster 20) that specifically expressed *IL1B*, *CD74*, *CD68*, and *HLA-DRA* and a subpopulation of H-type endothelial cells (EndC, Cluster 21) that highly expressed *PECAM1* and *EMCN* (Fig. 1e and Fig. 2c, d). To better understand the specific characteristics of hand chondrocytes, we compared transcriptomic differences between these chondrocyte subpopulations and knee chondrocytes.^20^ The results showed a close relationship of EC, RegC, ProC, preHTC, HTC and HomC in knee cartilage and hand cartilage (Fig. S2), which is consistent with the correlation results for cell subpopulations in hand cartilage only (Fig. S1h). In addition, FC in Ji et al.’s dataset showed notable differences from other subpopulations but exhibited the highest similarity to the FC observed in this study (Fig. S2).

### InflamC and FC are potentially key chondrocyte subpopulations in hand OA

**Fig. 3:**
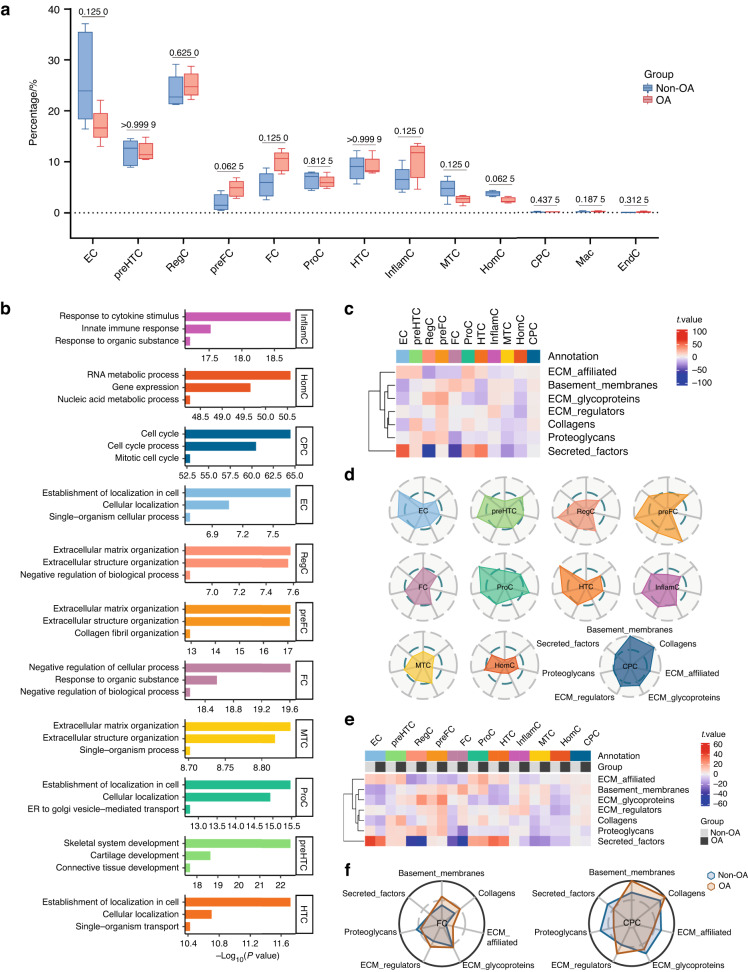
Hand OA-related alteration of proportion in subpopulations and their potential function. **a** Differential analysis of subpopulation abundance between hand osteoarthritic and nonosteoarthritic cartilage. The Wilcoxon matched-pairs signed rank test was used for analysis, and a *P* value < 0.05 was considered significant. **b** GO enrichment analysis of DEGs from each chondrocyte subpopulation, showing distinct potential functions in hand articular cartilage tissue. **c** and **d** Heatmap and radar map showing the performance of 7 matrisome gene sets among each chondrocyte subpopulation. **e** and **f** Heatmap and radar map showing evident changes in matrisome genes in FC and CPC between hand osteoarthritic and nonosteoarthritic status. OA osteoarthritis, EC effector chondrocytes, preHTC prehypertrophic chondrocytes, RegC regulatory chondrocytes, preFC prefibrocartilage chondrocytes, FC fibrocartilage chondrocytes, HTC hypertrophic chondrocytes, InflamC inflammatory chondrocytes, MTC mitochondrial chondrocytes, HomC homeostatic chondrocytes, CPC cartilage progenitor cells, DEGs differentially expressed genes, ECM extracellular matrix

Although no statistically significant differences were found between hand OA and non-OA cartilage, preFC, InflamC, and FC showed a trend toward increased numbers in the cartilage of hand OA joints compared with non-OA joint cartilage. In contrast, the proportion of EC, MTC and HomC tended to be lower in the cartilage of hand OA joints (Fig. 3a). As shown in Fig. 3b and Table S3, InflamC was found to play an exclusive role in the response to cytokine stimuli and the innate immune response. Marker genes of preFC and FC were preferentially enriched for the extracellular matrix (ECM) or structural organization. HomC was enriched in nucleic acid and RNA metabolic processes, consistent with a previous report of human knee osteoarthritic cartilage.^20^ Notably, compared to the other subpopulations, InflamC and FC were enriched in many inflammatory signaling pathways, e.g., reactive oxygen species pathway, TNFα signaling via NFκB, interleukin and interferon-mediated pathways (Fig. S3). Gene set variation analysis (GSVA) of matrisome genes revealed heterogeneous performance in ECM composition and regulation by distinct chondrocyte subpopulations (Fig. 3c, d). ECs, HTCs and ProCs preferentially expressed genes encoding secreted factors, whereas preFCs and RegCs tended to express ECM regulators and proteoglycans. Furthermore, CPC and FC seemed to undergo evident changes in ECM gene expression between nonosteoarthritic and osteoarthritic status, suggesting a potential role in hand OA (Fig. 3e, f).

**Fig. 4:**
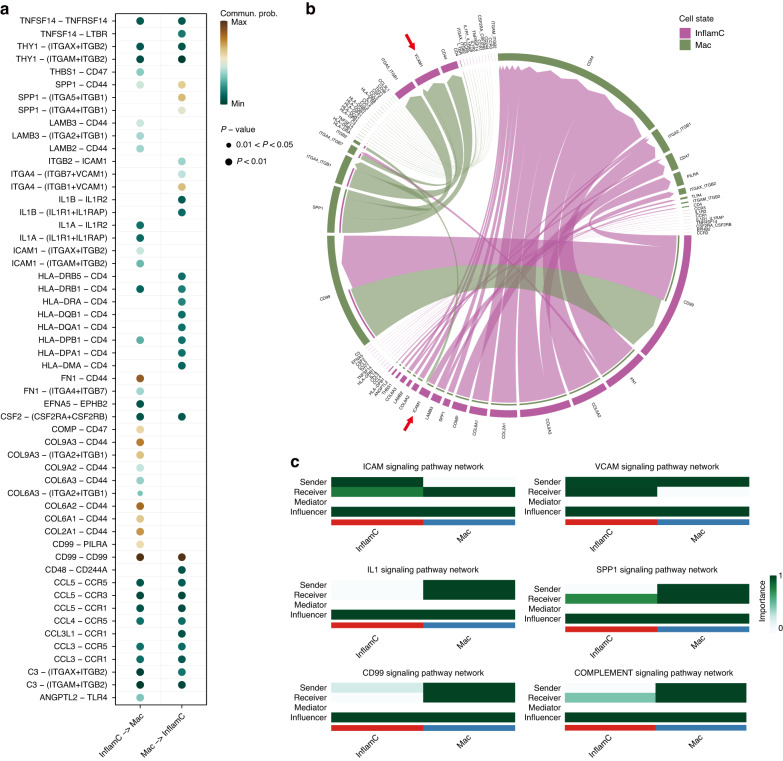
CellChat analysis of the interaction between InflamC and Mac. **a** Bubble plot showing the molecular pattern of interaction between InflamC and Mac. **b** Chord plot showing communication between InflamC and Mac. Arrows indicate *ICAM1* and *VCAM1* in intercellular communication. **c** Heatmap emphasizing the ICAM, VCAM, IL1, SPP1, CD99, and COMPLEMENT pathways activated by the InflamC-Mac interaction. InflamC inflammatory chondrocytes, Mac macrophages

After identifying the functional role of each subpopulation alone, we next sought to decode the pattern of intercellular communication using CellChat analysis. As expected, ECM-receptor communication was shown to occur among chondrocytes (Fig. S4a–d). We observed a dominant role of FC in Notch and platelet-derived growth factor pathways based on ligand‒receptor interactions with EndC, suggesting its potential role in angiogenesis associated with hand OA (Fig. S4e–h). Considering that InflamC specifically expressed genes related to the recruitment and proliferation of macrophages, we next focused on interaction between InflamC and Mac. *ICAM1* and *VCAM1*, marker genes of InflamC, were inferred to act on cellular contact and adhesion between InflamC and Mac (Fig. 4a, b). Furthermore, secretion of proinflammatory molecules such as *IL1B*, *SPP1*, *CD99*, and *C3* by Mac might act on their corresponding receptors on InflamC, probably eliciting downstream inflammatory processes (Fig. 4c). Collectively, our results support unique and cooperative roles for the identified cell subpopulations in maintaining hand articular cartilage homeostasis and suggest a potential role for InflamC and FC in hand OA.

### Ferroptosis is a key enriched pathway in hand osteoarthritic cartilage

**Fig. 5:**
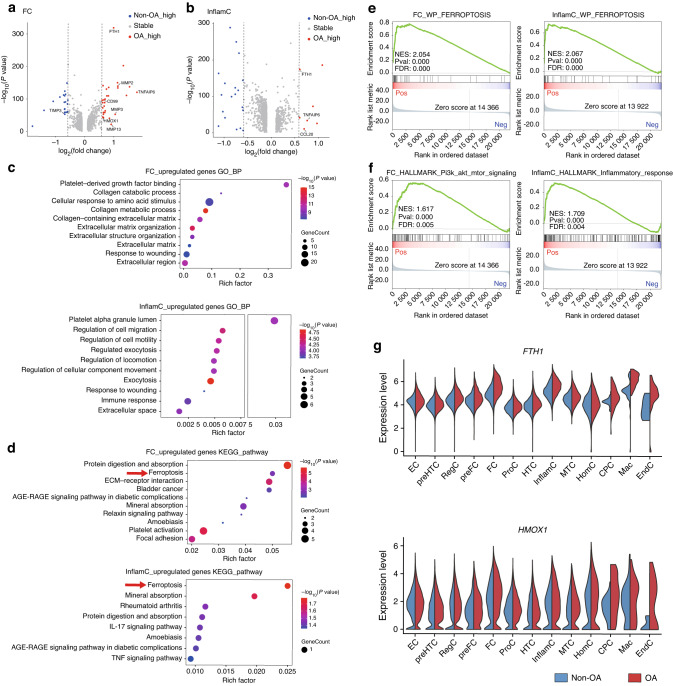
Alteration of expression patterns and enrichment of ferroptosis in hand OA. **a** Volcano plot showing DEGs of FC between hand osteoarthritic and nonosteoarthritic cartilage ( | log fold change | >0.5; *P* < 0.05). **b** Volcano plot showing DEGs of InflamC between hand osteoarthritic and nonosteoarthritic cartilage ( | log fold change | >0.5; *P* < 0.05). **c** GO enrichment analysis of upregulated genes of FC and InflamC in hand OA. **d** KEGG enrichment analysis of upregulated genes of FC and InflamC in hand OA. Arrows indicate ferroptosis enriched among upregulated genes in osteoarthritic FC and InflamC. **e** GSEA showing extensive enrichment of ferroptosis in hand osteoarthritic FC and InflamC. **f** GSEA showing extensive enrichment of PI3K-AKT-mTOR signaling and inflammatory response in hand osteoarthritic FC and InflamC, respectively. **g** Violin plot showing differential expression of *FTH1* and *HMOX1* in all subpopulations between hand osteoarthritic and nonosteoarthritic cartilage. OA osteoarthritis, DEGs differentially expressed genes, FC fibrocartilage chondrocytes, InflamC inflammatory chondrocytes, GO Gene Ontology, KEGG Kyoto Encyclopedia of Genes and Genomes, GSEA gene set enrichment analysis

Subpopulation-specific differences in gene expression between osteoarthritic and nonosteoarthritic cartilage are presented in Table S4. Given the potentially important role of InflamC and FC in hand OA, we focused on the difference between these two subpopulations. FC exhibited extensive alterations, with upregulation of a series of chondrocyte catabolic genes (e.g., *MMP2*, *MMP3*, and *MMP13*) and inflammatory response-related genes (e.g., *CCL20*, *CD99*, and *TNFAIP6*) in osteoarthritic cartilage (Fig. 5a). Seven genes, including inflammatory response-related genes (e.g., *CCL20* and *TNFAIP6*), were upregulated in InflamC from osteoarthritic cartilage (Fig. 5b). Gene Ontology (GO) analysis revealed that upregulated genes of FC in osteoarthritic cartilage were enriched in platelet-derived growth factor binding and collagen catabolic and metabolic processes and that upregulated genes of InflamC were enriched in regulation of cell migration and immune response (Fig. 5c). Furthermore, Kyoto Encyclopedia of Genes and Genomes (KEGG) analysis of osteoarthritic FC and InflamC indicated significant enrichment of many ECM-degrading pathways, such as protein digestion and absorption and mineral absorption (Fig. 5d). Notably, genes upregulated in InflamC and FC of hand osteoarthritic cartilage were both significantly enriched in the ferroptosis pathway (Fig. 5d).

We next conducted gene set enrichment analysis (GSEA) and identified significant enrichment of the ferroptosis gene set in both InflamC and FC in hand osteoarthritic cartilage, which highly suggests that ferroptosis plays a role in hand OA (Fig. 5e). In addition, genes related to the inflammatory response and the PI3K-AKT-mTOR pathway were highly enriched in InflamC and FC in osteoarthritic cartilage, respectively (Fig. 5f). DEG analysis of core ferroptotic genes revealed significant elevation in *FTH1* and *HMOX1* in FC, InflamC and Mac (Fig. 5g and Fig. S5). Notably, *FTH1* was consistently upregulated in both InflamC and FC, indicating iron overload in these cells during OA states (Fig. 5g). Furthermore, IHC analysis revealed that the protein level of FTH1 in human hand osteoarthritic cartilage was significantly elevated compared with that in nonosteoarthritic cartilage in the superficial and middle layers (Fig. S6). Together, these results demonstrate essential alterations in gene expression patterns in hand OA. Elevation of *FTH1* expression as well as enrichment of the ferroptosis pathway might represent a novel and vital molecular mechanism in hand OA.

### Comparative analysis of hand OA cartilage and knee OA cartilage reveal that *FTH1* and ferroptosis play key and unique roles in hand OA

Due to the observed differences in structure and stress environment, the cellular and molecular alterations occurring during hand OA and knee OA are expected to be different. Thus, we performed comparative analysis of our data and previous scRNA-seq data on knee OA.^20^ We first analyzed alteration of the cellular proportion in knee OA cartilage. The proportions of EC and HomC showed a significant decrease, whereas those of preHTC were significantly increased (Fig. S7). These results are consistent with observations for hand OA cartilage, in which EC and HomC showed a similar trend (Fig. 3a). However, FC, which exhibited an increasing trend in hand OA cartilage, was not significantly changed in knee OA. These results, together with the fact that InflamC was identified specifically in hand OA cartilage, represent a difference in cellular components between hand OA and knee OA (Fig. S7).

Subsequently, we compared DEGs in all cells during knee OA and hand OA. There were eight upregulated genes in hand OA cartilage, including *TNFAIP6*, *COL3A1*, *CCL20*, *FTH1*, *SERPINE2* and *VCAM1* (Fig. S8a), and 59 upregulated genes in knee OA cartilage, including *S100A4*, *COL3A1*, *HTRA1*, *TGFBI*, *OGN* and *ASPN* (Fig. S8b). GO enrichment analysis showed that genes involved in calcium-mediated signaling, regulation of cell migration, motility and locomotion were upregulated in hand OA cartilage (Fig. S8c). Genes involved in collagen and extracellular matrix organization were upregulated in knee OA cartilage (Fig. S8e). Notably, KEGG analysis revealed that the upregulated genes in both hand OA and knee OA were enriched in protein and mineral absorption and the AGE-RAGE signaling pathway (Fig. S8d); the ferroptosis pathway, which was identified as the key pathway in hand OA, was specifically enriched in hand OA cartilage (Fig. S8f). Intersection of the DEGs of knee OA and hand OA revealed seven shared DEGs, nine hand OA-specific DEGs, and 86 knee OA-specific DEGs (Fig. S8g). Functional analysis indicated the shared DEGs to be enriched in cell migration, motility and protein digestion and absorption (Fig. S8h, i). Notably, GO analysis showed that hand OA-specific DEGs were enriched in age-dependent responses to reactive oxygen species and oxidative stress (Fig. S8j). Moreover, KEGG analysis revealed that hand OA-specific DEGs were enriched in ferroptosis, in which *FTH1* was upregulated specifically in hand OA cartilage (Fig. S8k). Knee OA-specific DEGs were enriched in the extracellular matrix and protein digestion, consistent with previous studies (Fig. S8l, m).

As FCs were identified as key subpopulations in hand OA cartilage, we also performed DEG analysis for FCs between early-stage (stage 0 and 1) and late-stage (stage 3 and 4) knee OA cartilage. Upregulated genes of FC in late knee OA cartilage include *COL3A1*, *COL6A3*, *IFI27*, *IGFBP7*, *THY1* and *S100A10* (Fig. S9a). Functional enrichment analysis revealed that similar to hand OA cartilage, the upregulated genes of FC in late knee OA cartilage were enriched in extracellular matrix, extracellular vesicles, protein digestion and absorption (Fig. S9b, c). Intersection of the DEGs of FC in late knee OA and hand OA revealed nine shared DEGs, 47 hand OA-specific DEGs, and 502 knee OA-specific DEGs (Fig. S9d). Functional analysis showed the shared DEGs to be enriched in collagen catabolic processes, extracellular matrix organization and protein digestion and absorption (Fig. S9e, f). GO analysis showed that hand OA-specific DEGs and knee OA-specific DEGs were both enriched in the extracellular matrix and structure organization (Fig. S9g, i). Notably, KEGG analysis revealed that hand OA-specific DEGs were enriched in ferroptosis but that knee OA-specific DEGs were not. *FTH1*, which was identified as a key molecule of ferroptosis in hand OA, was uniquely and significantly upregulated in hand OA cartilage (Fig. S9h, j).

Taking all these results together, comparative analysis between hand OA cartilage and knee OA cartilage revealed that both cellular and molecular alterations during hand OA were quite different from those during knee OA. FC and InflamC, as well as the ferroptosis pathway, especially *FTH1*, play key and unique roles in cartilage degeneration in hand OA and might be specific targets for future intervention.

### MR analysis of the causal association between upregulated *FTH1* mRNA expression and the risk of hand OA in UK Biobank

Given that *FTH1* was consistently and significantly upregulated in InflamC and FC from osteoarthritic cartilage, we subsequently sought to investigate the causal association between *FTH1* mRNA expression and the risk of hand OA. We included 332 668 individuals of European descent from UK Biobank for MR analysis. The selection process of participants is presented in Fig. S10a, and the characteristics of the included individuals are presented in Table S5. Of the individuals, 2 418 (0.73%) individuals had a diagnosis of hand OA.

**Fig. 6:**
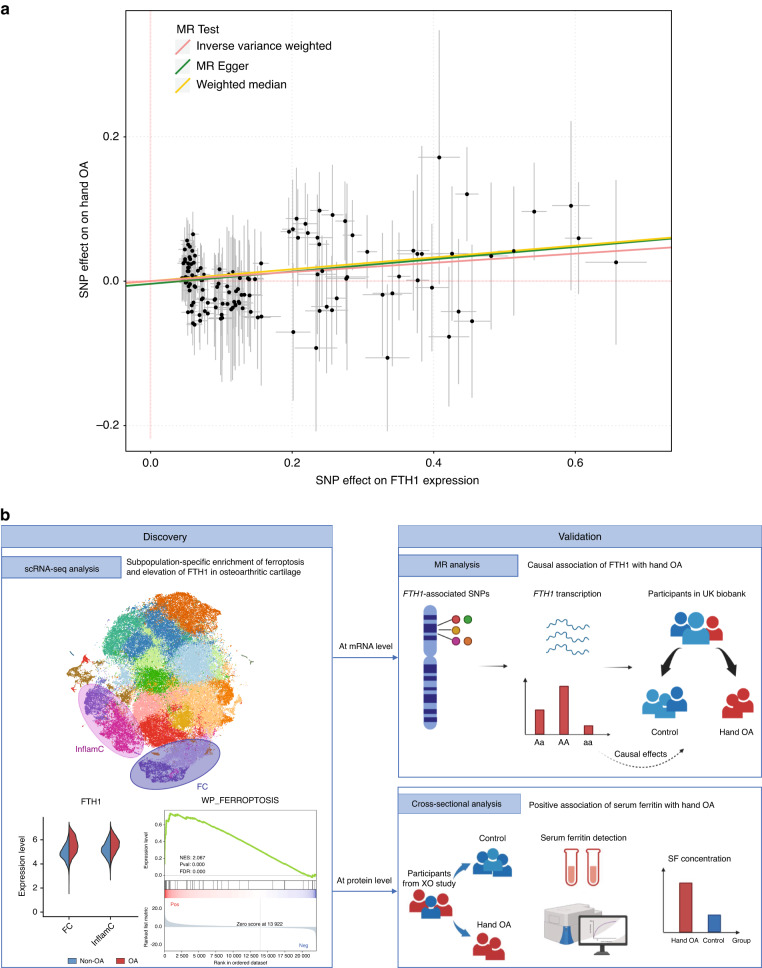
Mendelian randomization analysis of the causal association between *FTH1* mRNA expression and the risk of hand OA and cross-sectional analysis of the positive association between serum ferritin (encoded by *FTH1*) levels and the prevalence of hand OA. **a** Two-sample Mendelian randomization analysis of genetically predicted *FTH1* mRNA expression in hand OA. **b** Schematic illustration of this study. MR Mendelian randomization, OA osteoarthritis, SNP single-nucleotide polymorphism, XO study Xiangya Osteoarthritis Study

The selected genetic instrumental variables (Table S6) explained 65.6% of the variance in *FTH1* mRNA expression. Univariate two-sample MR analysis showed that a genetic predisposition toward higher expression of *FTH1* mRNA significantly increased the risk of hand OA (odds ratio [OR] = 1.07 per standard deviation [SD] increase in *FTH1* expression, 95% confidence interval [CI]: 1.02–1.11, *P* = 0.005) (Table 1). Moreover, the direction of the effect estimate was consistent across the four MR methods assessed (i.e., inverse-variance weighted [IVW], MR‒Egger, weighted median, and MR-PRESSO) (Fig. 6a and Table 1). We did not observe heterogeneity in the present analysis (*P*_*Cochran’s Q*_ = 0.999), and MR‒Egger intercepts indicated limited evidence of directional pleiotropy (*P* = 0.491).

**Table 1:** The effect of genetically predicted *FTH1* mRNA expression on hand OA

Exposure	N SNPs	MR Method	OR (95% CI)	*P* value
*FTH1* mRNA expression	133	IVW	1.07 (1.02–1.11)	0.005
		Weighted median	1.09 (1.00–1.18)	0.046
		MR Egger	1.08 (1.00–1.18)	0.046
		MR-PRESSO	1.07 (1.02–1.11)	0.006

*OA* osteoarthritis, *N SNPs* number of single-nucleotide polymorphisms, *IVW* inverse-variance weighted, *MR* Mendelian randomization, *OR* odds ratio, *CI* confidence interval

### Serum ferritin level and prevalence of hand OA in Xiangya Osteoarthritis Study

Ferroptosis is a form of regulated cell death initiated by perturbations of the intracellular microenvironment, which relies on iron availability.^25^ Our current findings, e.g., enrichment of the ferroptosis pathway and upregulated expression of *FTH1* mRNA in osteoarthritic cartilage by scRNA-seq analysis and a causal association between upregulated *FTH1* mRNA expression and the risk of hand OA by MR analysis, prompted us to investigate whether iron load is elevated in patients with hand OA. For this purpose, we measured serum ferritin, a biomarker for body iron stores,^26^ in Xiangya Osteoarthritis Study participants.^18,19^ A total of 1 241 participants were included (50.9% were female, mean age 62.8 years). The selection process is outlined in Fig. S10b. Of these participants, 392 (31.6%) had hand OA. The baseline characteristics according to hand OA status are shown in Table S7.

As indicated in Table 2, compared with the lowest quintile of serum ferritin, the crude ORs for hand OA were 1.07 (95% CI: 0.74–1.56), 1.07 (95% CI: 0.74–1.56), 1.27 (95% CI: 0.88–1.84), and 1.55 (95% CI: 1.08–2.22) in the second, third, fourth and highest quintiles of serum ferritin, respectively (*P*-for-trend = 0.037). Adjustment for age, sex and BMI did not change the results materially (*P*-for-trend = 0.005).

**Table 2:** Association between serum ferritin levels and the prevalence of hand OA

Items	Quintiles of serum ferritin /(μg·L^−1^)					*P*-for-trend
	Q1	Q2	Q3	Q4	Q5	
Hand (*n*)^a^	495	496	491	494	487	
Median of serum ferritin levels/(μg·L^−1^)	75.0	138.5	213.0	308.0	564.0	
Number of hand OA/%	99 (20.0)	105 (21.2)	104 (21.2)	119 (24.1)	136 (27.9)	
Crude OR (95% CI)	1.00 (Ref)	1.07 (0.74–1.56)	1.07 (0.74–1.56)	1.27 (0.88–1.84)	1.55 (1.08–2.22)	0.037
Age-sex-BMI adjusted OR (95% CI)	1.00 (Ref)	1.31 (0.89–1.93)	1.23 (0.83–1.83)	1.46 (0.97–2.22)	1.69 (1.13–2.52)	0.005

*OA* osteoarthritis, *Q* quantile, *n* number, *OR* odds ratio, *CI* confidence interval, *BMI* body mass index, *Ref* reference

^a^The association between serum ferritin levels and hand osteoarthritis was evaluated by a logistic regression model using generalized estimating equations (hand-specific analysis)

## Discussion

Mounting evidence points to a distinctive pathophysiology of hand OA compared with knee or hip OA.^4,5^ Unlike the large weight-bearing knee or hip joints, the pathophysiology of hand OA remains largely unknown, partially because of limited access to clinical sample tissues and a lack of animal models.^2^ In this study, for the first time, we applied scRNA-seq technology to identify chondrocyte subpopulations and investigate the molecular mechanism of hand OA. We provide a transcriptomic atlas of 105 142 cells from paired hand articular cartilage samples of osteoarthritic and nonosteoarthritic interphalangeal joints. InflamC and FC were identified as key subpopulations in hand OA. In both subpopulations, the ferroptosis pathway was enriched, and expression of iron overload-related genes, e.g., *FTH1*, was elevated. Moreover, we validated the gene (i.e., *FTH1*) and pathway (i.e., ferroptosis) identified by scRNA-seq through MR analysis involving 332 668 individuals from UK Biobank and a cross-sectional analysis of 1 241 participants in Xiangya Osteoarthritis Study,^18,19^ respectively. Figure 6b shows a schematic illustration of this study.

We identified 21 putative clusters that were annotated into 13 cell subpopulations according to the markers from previous scRNA-seq studies of knee articular cartilage and meniscus.^20–24^ In addition, we identified a novel and unique chondrocyte subpopulation (InflamC) that expressed proinflammatory genes such as *CCL2*, *CCL20*, *ICAM1*, *NOS2*, and *TNFAIP2*. It is widely appreciated that chronic, low-grade inflammation contributes to OA symptomatology and progression.^27^ Inflammatory mediators can alter chondrocyte differentiation and function and activate cartilage-degrading enzymes.^28^ Moreover, we observed intimate communication between subpopulations InflamC and Mac. A pattern of InflamC-mediated macrophage recruitment by chemokines and adhesion molecules and Mac-mediated local inflammation by inflammatory cytokines was thus hypothesized as contributing to hand OA, though experimental validation is needed. Furthermore, the proportion of InflamC in hand osteoarthritic cartilage tended to be higher than that in nonosteoarthritic counterparts. Altogether, our data suggest a crucial role of InflamC in development of hand OA. Future studies on this unique chondrocyte subpopulation and its marker genes may be valuable for drug discovery and development.

This large dataset also enabled discovery of rare cells and their transcriptomic profiles. CPC, Mac and EndC were identified in hand articular cartilage, together comprising only <0.5% of 105 142 cells. Previous research has shown that articular cartilage has a low intrinsic capacity for self-repair.^29^ In our dataset, marker genes of CPC were enriched specifically for the cell cycle and mitosis, suggesting potential roles in chondrocyte regeneration and cartilage repair. Although it is often considered that articular cartilage consists of only chondrocytes embedded in the ECM,^28^ our data suggest that a very small subpopulation of macrophages and endothelial cells exist in hand articular cartilage. Recent evidence has shown vascular invasion into both calcified and even noncalcified cartilage from the subchondral bone during OA. Nevertheless, the frequency and area of vascular invasions into cartilage are relatively small.^30–32^ Furthermore, bioinformatic analysis of scRNA-seq data of human knee cartilage revealed a very minimal number of macrophages and endothelial cells.^33^ Ji et al.’s analysis also identified a small proportion of cells expressing CD74 and CD86, indicating their antigen-presenting and processing function in immune cells.^20^ Overall, our discovery of rare cells, including macrophages and endothelial cells, aligns with these previous findings. Macrophages might secrete cytokines into the joint microenvironment, resulting in a flare of local inflammation that amplifies and perpetuates cartilage degradation. H-type vessels are characterized by high expression of *CD31* and endomucin (*EMCN*), which were both detected in EndC in our data. Evidence indicates that H-type endothelial cells couple angiogenesis and osteogenesis, leading to subchondral bone remodeling and cartilage matrix degradation in OA.^34^ Although macrophages and H-type endothelial cells comprise a small proportion of total cells, their potential function, particularly their interaction with InflamC and FC, might contribute to the vascular invasion and inflammatory response in hand OA cartilage. Therefore, our data, together with previous studies, indicate that although nonchondrocytes, i.e., macrophages and endothelial cells, account for a small proportion of hand OA cartilage, they might play a direct and important role in progression of hand OA. Together, the small cell subpopulations discovered in our data might have a vital role in the pathogenesis of hand OA and serve as targets for future translational research.

Another key finding of this study was extensive alteration of gene expression patterns in FC in hand OA. Loss of chondrocyte phenotypic stability constitutes a hallmark event during OA progression.^28^ FC was characterized by expression of fibroblast markers (e.g., *COL1A1* and *COL1A2*), but chondrocyte markers of hyaline cartilage (e.g., *COL2A1* and *ACAN*) were rarely expressed. FC was previously shown to play an active role in vasculature development and endochondral ossification and to dominate in late-stage osteoarthritic cartilage.^20^ In our dataset, FC contributed less to ECM secretion and regulation and showed active interaction with endothelial cells. More intriguingly, when comparing DEGs between osteoarthritic and nonosteoarthritic cartilage, cartilage-degrading enzymes (*MMP2*, *MMP3*, and *MMP13*) were highly upregulated, whereas a metallopeptidase inhibitor (*TIMP3*) was significantly downregulated. In summary, FC, in close correlation with increased breakdown and impaired repair of cartilage, was largely expanded and appears to be another target cell subpopulation in hand OA.

Enrichment of ferroptotic genes was observed in hand osteoarthritic cartilage. Ferroptosis, a distinct cell death pathway characterized by iron-dependent phospholipid peroxidation, has recently been demonstrated to have a pathological role in various conditions.^35^ Our discovery revealed that subpopulations FC and InflamC in hand osteoarthritic cartilage consistently exhibit enrichment for genes involved in ferroptosis, among which *FTH1*, a protein-coding gene for ferritin, is significantly upregulated. Ferritin is a major cellular iron storage protein and has a crucial role in maintaining cellular iron homeostasis.^26^ Our findings suggest an increase in the molecular response to intracellular iron in excess resulting from an attempt to reduce the iron labile pool in osteoarthritic FC and InflamC.^36^ Moreover, previous genetic studies have revealed that genetic variants of *HFE*, which encodes a homeostatic iron regulator, are associated with hand OA.^37^ These results indicate that dysregulation of iron homeostasis and ferroptotic cell death are involved in hand OA.

We conducted two independent large-sample population-based studies at the mRNA and protein levels separately to validate our hypothesis. MR analysis is a well-established and commonly used genetics technique to estimate the causative effect of an exposure variable on an outcome while minimizing the risk of confounding and reverse causation.^17^ Based on data from a large expression quantitative trait locus (eQTL) analysis and UK Biobank, we found a causal association between upregulated *FTH1* mRNA expression and increased risk of hand OA. Elevated serum ferritin levels have been implicated as a marker of iron overload and are associated with multiple diseases.^38,39^ By using data from another community-based study in China (i.e., the Xiangya Osteoarthritis Study^18,19^), we found that a high serum ferritin level was positively associated with a higher prevalence of hand OA. Taken together, these findings support the contribution of iron homeostasis and ferroptosis to hand OA, shedding light on a new molecular mechanism and potentially promising therapeutic target for hand OA. Ferroptosis inhibitors or iron chelators, such as ferrostatin-1^40^ and deferoxamine,^41^ are thus potential treatments for hand OA, and experimental validation is warranted.

Several strengths of our study are noteworthy. We provide the first description of hand articular cartilage subpopulations at single-cell resolution. In addition, based on the results from scRNA-seq analysis that revealed enrichment of ferroptosis and elevation of iron overload-related genes in osteoarthritic cartilage, we validated this association of ferritin, as measured at the mRNA and protein levels, with hand OA in two independent population-based studies. Because *FTH1* mRNA expression was instrumented using randomly allocated single-nucleotide polymorphisms (SNPs), MR analysis findings are not associated with reverse causation and minimize potential confounding biases. By using data collected from Xiangya Osteoarthritis Study, we showed a positive relationship between serum ferritin levels and the prevalence of hand OA. This result corroborates the findings from scRNA-seq analysis and MR analysis, suggesting the robustness of our study results.

The limitations of this study include a relatively small sample of hand osteoarthritic and nonosteoarthritic cartilage samples. Another limitation was that we were unable to validate our findings in vivo due to the lack of an animal model for hand OA.^2^ Third, as hand OA is a whole-joint condition, future studies may benefit from the addition of other joint components (such as the synovium, subchondral bone and synovial fluid) for combined and in-depth decoding of hand OA development.

In summary, we generated molecular atlases of hand articular cartilage at single-cell resolution. Our findings suggest that specific subpopulations of chondrocytes, namely, InflamC and FC, play a role in hand OA. The molecular profiles (e.g., *FTH1* and serum ferritin) and their related pathways (e.g., ferroptosis) presented will inform future therapeutic strategies for hand OA.

## Materials and methods

### ScRNA-seq sequencing

#### Participant recruitment

Intact hand interphalangeal joint tissue samples were obtained from five donors who underwent amputations due to severe destructive injuries of the forearm. Clinical information was collected from medical records (Table S1). Subjects were excluded if they had or previously had any other osteoarticular disease (such as rheumatoid arthritis or gout) or disorder of osteochondrodysplasia. All donors provided informed consent, which was approved by the Ethics Committee of Xiangya Hospital, Central South University, China (IRB number: 202108350).

#### Sample collection

The hands of the donors were washed three times with 75% ethanol and stored in a sterile bag. After transport to the cell culture facility, the hands were washed three times with 75% ethanol, and the joint capsules were opened under sterile conditions.

Each of the interphalangeal joints from each donor’s hand was diagnosed as an osteoarthritic joint (stage II–IV) according to Modified Outerbridge Classification^42,43^ or a nonosteoarthritic joint (stage 0) based on the macroscopic appearance (Fig. 1b). We obtained two specimens of whole articular cartilage from the same donor, i.e., one from the osteoarthritic interphalangeal joint and another from the nonosteoarthritic interphalangeal joint, and subjected them to scRNA-seq. Because nonosteoarthritic and osteoarthritic cartilage from each person formed a matched set, the effects of all person-level confounders (such as age, sex, body mass index) were implicitly eliminated.

#### Single-cell suspension preparation

Osteoarthritic and nonosteoarthritic cartilage specimens were harvested from each donor and subjected to chondrocyte isolation according to a previously described two-step digestion protocol.^24^ Briefly, minced cartilage slices were immersed in 2 mg·mL^−1^ pronase (Millipore, USA) in Dulbecco’s modified Eagle’s medium/nutrient mixture F-12 (DMEM/F12) (Thermo Fisher Scientific, USA) and incubated for 60 min at 37 °C. The pronase solution was then removed and replaced with DMEM/F12 containing 0.36 mg·mL^−1^ collagenase P (Roche Diagnostics GmbH, Germany) and 10% fetal bovine serum (Sigma‒Aldrich, USA) for 12 h at 37 °C in an atmosphere of 5% CO_2_. Undigested debris was removed by filtration through a 70 μm cell strainer. The cell suspensions were pelleted by centrifugation, washed with sterile phosphate-buffered saline (PBS) and used immediately for scRNA-seq library construction (10× Genomics, USA).

#### Cell type annotation

The identities of each cluster were annotated based on known marker genes in previously published articles,^20–24^ and clusters were considered the same subpopulation based on shared marker genes. Briefly, 12 previously reported subpopulations were identified: (1) EC, which highly expressed *CYTL1*, *CHAD* and *FRZB*; (2) preHTC, as marked by simultaneously high expression of *COL2A1, COL10A1*, *COL11A1* and *COL11A2*; (3) RegC, which featured expression of *CHI3L1*, *CRTAC1*, *CLU* and *CHI3L2*; (4) preFC, which showed high levels of *HTRA1* and *OGN*; (5) FC, which demonstrated high expression of *COL1A1*, *TNFAIP6* and *COL1A2*; (6) ProC, which highly expressed *P3H2*, *UPP1* and *LRRFIP1*; (7) HTC, as marked by high levels of *COL10A1*, *SPP1* and *IBSP*; (8) MTC, expressing high levels of *MT-CYB* and *MT-CO3*; (9) HomC, as identified by the markers *JUN*, *HES1*, *MT1X* and *MT1E*; (10) CPC, showing specific *STMN1* and *PTTG1* expression; (11) Mac, as marked by distinct expression of *IL1B*, *CD74*, and *CD68*, *HLA-DRA*; and (12) EndC, with high expression of *PECAM1*, *EMCN*. One novel chondrocyte subpopulation was annotated according to its distinct expression of *CCL20*, *CCL2*, *NOS2* and *MMP3*, as confirmed by significant enrichment in gene sets of inflammatory response, immune system process and immune response (Fig. 1d, e). We also conducted comparative analysis to confirm the similarities between chondrocyte subpopulations from human hand cartilage in this study and human knee cartilage from Ji et al. (GEO: GSE104782).^20^ We calculated the Pearson correlation of the average expression of genes in cell subpopulations between hand and knee cartilage.

#### Cellular composition analysis

We calculated the proportion of cell subpopulations in each cartilage specimen using the number of cells from a specific cell subpopulation divided by the total number of cells and compared the proportion of each specific cell subpopulation between osteoarthritic cartilage specimens and their matched nonosteoarthritic cartilage specimens using the Wilcoxon matched-pairs signed rank test. *P* values < 0.05 were considered significantly different.

#### Functional analysis of DEGs

For functional annotation, enrichment with GO biological processes was performed on DEGs using the R package topGO (v2.44.0).^44^ Only terms with *P* < 0.05 were retained. GSVA was performed with hallmarks^45^ or matrisome gene sets^46^ to identify distinct functional roles or ECM expression patterns among subpopulations. The ggradar package (v0.2) was used to visualize the relative abundance of a given gene set in each chondrocyte subpopulation.

DEG analysis for osteoarthritic versus nonosteoarthritic cells for each cluster was performed using the Wilcoxon rank-sum test in the “FindMarkers” function in Seurat, with a cutoff of *P* < 0.05 and fold change (FC) > 1.5. For functional annotation, GO and KEGG enrichment analyses were performed on the DEGs. The top 10 significant terms were visualized by a bubble plot. GSEA was performed using the GSEApy Python package (0.10.4).^47^ The hallmark and ferroptosis pathway gene sets were downloaded from the Molecular Signatures Database (7.5.1). The statistical significance (nominal *P* value) of the enrichment score was calculated by using an empirical phenotype-based permutation test. A normalized enrichment score (NES) was obtained, and the false discovery rate corresponding to each NES was calculated.

#### CellChat analysis

To identify potential intercellular interactions, we performed CellChat analysis^48^ according to the standard pipeline. To obtain biologically significant cell‒cell communications, probability values for each interaction were calculated, and permutation tests were performed. The inferred intercellular communication network of specific signaling pathways was visualized by circle plots. We also computed the importance of each cell cluster and used measures in weighted-directed networks to identify dominant senders, receivers, mediators and influencers for intercellular communications. We visualize all significant interactions (L-R pairs) from InflamC/Mac to Mac/InflamC using bubble and chord plots.

#### IHC

Freshly dissected human hand joint tissues were fixed for 5 days in 4% paraformaldehyde, decalcified in 15% ethylenediamine tetraacetic acid (EDTA) and embedded in paraffin for sectioning. Serial sections (5 μm thick) were obtained, deparaffinized in xylene and then rehydrated in decreasing concentrations of ethanol. The streptavidin-biotin system (ZSGB-BIO, CN) was used following the manufacturer’s recommended protocol. After blocking with 10% normal goat serum for 15 min at room temperature, the sections were incubated with anti-ferritin heavy chain (FTH1) (Abcam, 1:200, ab75972), anti-inducible nitric oxide synthase (iNOS) (Abcam, 1:1 000, ab178945), anti-macrophage inflammatory protein 3 alpha (MIP-3α) (Abcam, 1:500, ab224188), anti-interleukin-1 beta (IL-1β) (Abcam, 1:500, ab283818), anti-pituitary tumor-transforming gene 1 protein (PTTG1) (Abcam, 1:250, ab79546), and/or anti-platelet endothelial cell adhesion molecule (CD31) (Abcam, 1:250, ab76533) antibodies overnight at 4 °C. Subsequently, the sections were washed with PBS and incubated with a secondary biotinylated goat anti-rabbit antibody for 15 min followed by streptavidin for 15 min. Diaminobenzidine tetrahydrochloride chromogen substrate solution was then added to stain the sections, and hematoxylin counterstaining was performed. All IHC staining was assessed by light microscopy. The articular cartilage was divided into three zones according to light microscopy observations: superficial, middle and deep.^49^

### Comparative analysis of hand OA cartilage and knee OA cartilage

We downloaded metadata and processed cell-gene matrix data from the GEO database (GSE104782) with author-based annotations.^20^ For comparison of cellular alterations between hand OA and knee OA, we first calculated the proportion of cell subpopulations in each knee cartilage specimen. We compared the proportion of each specific cell subpopulation between knee osteoarthritic cartilage specimens (stages 1–4) and their matched nonosteoarthritic cartilage specimens (stage 0) using the Wilcoxon matched-pairs signed rank test. In addition, as Ji et al. grouped the cells into two stages, i.e., early-stage (stage 0 and 1) and late-stage (stage 3 and 4) in their study, we compared the proportion of each specific cell subpopulation between early-stage and late-stage knee osteoarthritic cartilage. *P* values < 0.05 were considered significantly different.

For comparison of molecular alterations between hand OA and knee OA, we performed volcano plot visualization of gene expression between the cells in hand OA and non-OA cartilage in our dataset. Similarly, using Ji et al.’s dataset, we performed volcano plot visualization of gene expression between the cells in knee OA (stage 1–4) and non-OA (stage 0) cartilage. We performed GO and KEGG enrichment analyses of upregulated genes in hand OA and knee OA, respectively. Subsequently, we intersected DEGs of knee OA and hand OA and grouped the genes as shared DEGs of knee and hand OA, hand OA-specific DEGs, and knee OA-specific DEGs. GO and KEGG enrichment analyses were performed to explore the function of these groups of genes. We also performed DEG analysis for FC between early-stage (stage 0 and 1) and late-stage (stage 3 and 4) knee OA cartilage, but DEG analysis between OA (stage 1–4) and non-OA (stage 0) was not applicable because a very small number of FCs was detected at stage 0. GO, KEGG and intersection analyses were conducted as described above.

#### Statistical analysis

ScRNA-seq analysis was performed using R 4.0.2, Python 3.8.6, and SPSS 18.0. The proportion of each specific cell subpopulation between osteoarthritic cartilage specimens and their matched nonosteoarthritic cartilage specimens was determined using the Wilcoxon matched-pairs signed rank test. DEG analysis was performed using the Wilcoxon rank-sum test. For IHC analysis, one-way repeated measures analysis of variance (ANOVA) was used to compare the percentages of positive cells in each cartilage zone.

### Two-sample MR study for the association between *FTH1* and hand OA

We performed two-sample MR analysis using the summary-level genetic data retrieved from an expression quantitative trait locus (eQTL) study^50^ and individual-level data from UK Biobank.^51^ Hand OA was defined by physician diagnosis using International Classification of Diseases, Ninth Revision codes or Tenth Revision codes. Corresponding β coefficients and standard errors of the associations between *FTH1*-associated single-nucleotide polymorphisms (SNPs) and hand OA were calculated using logistic regression while adjusting for age, sex, genotype measurement batch and 20 genetic principal components. We obtained the odds ratio (OR) of the risk of hand OA prevalence for one standard deviation (SD) in the expression level of *FTH1* using inverse-variance weighted (IVW) meta-analysis with a multiplicative random-effects model. We conducted three sensitivity analyses (i.e., weighted median, MR‒Egger and MR Pleiotropy RESidual Sum and Outlier [MR-PRESSO] methods) to examine the presence of horizontal pleiotropy and address the detected heterogeneity.^52,53^ We also examined the heterogeneity of SNP effects using Cochran’s test.^54^

### Cross-sectional study of association between serum ferritin and hand OA

We evaluated the association between serum ferritin levels and hand OA prevalence (determined by radiographic assessment) among participants in Xiangya Osteoarthritis Study subcohort I.^18,19^ We performed generalized estimating equations (GEEs) (hand-specific analysis) with logit links to obtain ORs and 95% confidence intervals (CIs) of prevalent hand OA for each quintile category of serum ferritin levels.^55^ In the multivariable regression model, we adjusted for age, sex and BMI.

For more details about the study design and statistical methods, see Supplementary Methods.

### Supplementary information

Supplementary Data

Dataset 1

## Data Availability

All data analyzed during this study are included in this published paper and its supplementary information files. The raw sequence data reported in this paper have been deposited in the Genome Sequence Archive (Genomics, Proteomics & Bioinformatics 2021) in National Genomics Data Center (Nucleic Acids Res 2022), China National Center for Bioinformation/Beijing Institute of Genomics, Chinese Academy of Science (GSA-Human: HRA005525) that are publicly accessible at https://ngdc.cncb.ac.cn/gsa-human. Additional data related to this paper are available from the corresponding author upon reasonable request.
