# Supplementary material for: Combining single-cell RNA sequencing and population-based studies reveals hand osteoarthritis-associated chondrocyte subpopulations and pathways
Source: Bone Res. 2023 Nov 2;11:58. doi: 10.1038/s41413-023-00292-7 (PMC10620170; doi:10.1038/s41413-023-00292-7)
Supplement: Supplementary file 1 — Supplementary Data [file 41413_2023_292_MOESM1_ESM.docx]

Supplementary Data

**This Word file includes:**

Supplementary Methods

Supplementary Fig. S1 to S11

**Supplementary Methods**

**ScRNA-seq data processing**

***ScRNA-Seq Library Preparation and Sequencing***

For the quality check and counting of single cell suspension, the cells were resuspended to prepare a suitable cell concentration of 700~1200 cells/μL and the cell survival rate is generally above 90%. Cells were counted and loaded onto the Chromium Controller (10X Genomics, USA) (Single-Cell 3’ Library and Gel Bead Kit v.3). Generation of gel beads in emulsion (GEMs), barcoding, GEM-RT clean-up, and complementary DNA were all performed as per the manufacturer’s protocol. Qubit was used for library quantification before pooling. The final library pools were sequenced on the Illumina Novaseq 6,000 platform using 150-base-pair paired-end reads.

***Alignment and Quantification***

Raw scRNA-seq data were processed, examined by quality metrics, mapped to the pre-built human reference genome (GRCh38) and assigned to individual cells of origin according to the cell specific barcodes, using the Cell Ranger pipeline (v6.0.1, 10X Genomics). In total, 108,062 single cell transcriptomes with 2,535 median genes per cell were generated for further quality control and analysis (**Supplementary Table S8**).

***Quality Control and Clustering***

Cell-gene Unique Molecular Identifier (UMI) counting matrices were subjected to Scrublet in Python (v3.7.3) ^1^ for identifying and removing doublets. For clustering and cell type identification, the Seurat R package (v3.2.0) ^2,3^ was used. Specifically, we examined the data and performed further filtering (nFeature_RNA>200, percent_mt<10, percent_redcell<10). We aligned the individual objects for canonical correlation analysis to minimize batch effects for integrated analysis.

Datasets were processed with Principal Component Analysis (PCA) using the 3000 most variable genes as input, followed by clustering with louvain algorithm using the top 30 principal components and setting the resolution parameter to 0.9 for fine clustering. T-Stochastic Neighbor Embedding (t-SNE) ^4^ was adopted for subsequent dimensional reduction and visualization. Marker genes of each cluster were identified using the Seurat function “FindAllMarkers” with the following filtering standards: (1) log2 fold-change of the average expression between the putative cluster and other clusters>0.58; (2) the percentage of cells expressing the gene in the cluster >25%; (3) the percentage of cells expressing the gene in the cluster > the percentage of cells expressing the gene in other clusters; (4) adjusted *P* value<0.05. One cluster with considerably smaller total UMI counts were assumed to consist of nuclei that had been stripped of their cytoplasm in 10x droplets. One cluster with abnormally high UMI counts but no strongly cluster-specific genes were considered as a technical artefact of cell doublets, and confirmed to have a high proportion of detected doublets (doublet score>0.25) by Scrublet. These two clusters were therefore excluded from downstream analysis according to the established methods ^5^.

**MR study**

***Study Design***

MR analysis is a well-established and commonly-used genetics technique to estimate and test for the causative effect of an exposure variable on an outcome, while minimizing the risk of confounding and reverse causation ^6,7^. We performed a two-sample MR using the summary-level genetic data retrieved from an eQTL study ^8^ and the individual-level data from the UK Biobank ^9^. Ethics approval for the UK Biobank study was obtained from the North West Centre for Research Ethics Committee (11/NW/0382).

***Genetic Instruments***

We obtained the cis-eQTLs data derived from whole blood for *FTH1* mRNA expression from the eQTLGen project ^8^. To guarantee statistical independence, SNPs with the pairwise linkage disequilibrium (LD) R^2^>0.9 were excluded ^10^. We further removed SNPs (eQTLs) with minor allele frequency (MAF) <0.01, and finally 133 significant SNPs (*P*<5×10^-8^) were selected for subsequent analysis. Because the effect sizes of the cis-eQTLs were not available in the summary data, we estimated them from z statistics as previously described ^10^. The SNPs used as instruments of exposure are presented in **Supplementary Table S6**.

***Outcome Definition***

Individual-level phenotypic data on hand OA was retrieved from the UK Biobank, which is a prospective cohort containing data of 500,000 adults aged 40-69 years in the United Kingdom and enrolled between 2006 and 2010. The study protocol was available online and more details were published elsewhere (Application No. 77,646) ^9^. We excluded participants who had non-white European ancestry (for the purpose of minimizing confounding by ancestry), sex mismatches, excess heterozygosity, missingness, or closer than 3rd degree relatives. Hand OA was defined by hospital diagnosis using the International Classification of Diseases, Ninth Revision codes or the Tenth Revision codes as previous described (**Supplementary Table S9**) ^11^.

***Statistical Analysis***

We firstly calculated the β coefficients and standard errors for the associations between *FTH1* associated-SNPs and hand OA using logistic regression, adjusting for age, sex, genotype measurement batch and 20 genetic principal components. Then we performed the IVW meta-analysis with a multiplicative random-effects model to calculate the ORs with their 95% CIs calibrated to the effect of one SD increase in the causal association of *FTH1* with hand OA. We conducted three sensitivity analyses (i.e., Weighted median, the MR-Egger and the MR-PRESSO methods) to examine the presence of horizontal pleiotropy and address the detected heterogeneity ^12,13^. We also examined the heterogeneity of the SNP effects using the Cochran’s test ^14^. Two-sample MR analyses were implemented with the MendelianRandomization package and MR-PRESSO package in R (version 3.6.3). The associations of genetic variants with hand OA were conducted via the PLINK (version 2.0) software.

**Cross-sectional study**

***Study Participants***

The participants included in this study were retrieved from the Xiangya OA Study, which is a community-based longitudinal study focusing on the natural history and risk factors of OA in a rural area of China (NCT04033757) ^15,16^. Participants aged ≥50 years were randomly selected from mountainous villages of Longshan County in Hunan Province in the following steps: 1) 14 communities were selected from the 46 communities in Longshan County by the probability proportionate to size sampling method; 2) all the villages in the 14 communities were listed in a random order; 3) village-to-village recruitment was repeated beginning with the first village in the first community until the number of subjects in that community reached the pre-determined quota set by the Sixth National Census Data of Longshan County (2010). Eventually, 25 mountainous villages in Longshan County were covered. The Xiangya OA Study consists of three sub-cohorts (i.e., sub-cohort Ⅰ, Ⅱ and Ⅲ), which were initiated in 2015, 2018 and 2019, respectively. The Xiangya OA Study was conducted with the approval of the medical ethical committee at Xiangya Hospital (201510506). Informed consent was obtained from all participants.

Participants included in the present study were selected from sub-cohort Ⅰ. Among the 1,469 age-eligible (age≥50 years) residents of Longshan County who were randomly selected from the sub-cohort Ⅰ, 228 were excluded due to: (1) unavailability of questionnaire data (n=3); (2) unavailability of radiographs of hand, (n=78); (3) unavailability of serum ferritin data (n=97); (4) participants with rheumatoid arthritis (n=50). Finally, 1,241 participants were included (**Supplementary Table S7**).

***Measurement of Serum Ferritin Concentration***

Blood samples were drawn after 12 hours of fasting and stored at 4 ℃ until testing. The serum ferritin concentration was measured using the chemiluminescence method by Beckman Coulter DXI800. The inter-assay coefficients of variation were 4.1% (37.2 ng/mL), 4.3% (118.9 ng/mL) and 6.3% (311.8 ng/mL) for serum ferritin. The intra-assay coefficients of variation were 2.6% (37.2 ng/mL), 3.6% (118.9 ng/mL) and 3.9% (311.8 ng/mL) for serum ferritin ^17^.

***Assessment of Hand OA***

Participants in the Xiangya OA Study underwent a posterior-anterior (PA) radiograph of both hands. Radiographs of the bilateral second to fifth distal interphalangeal (DIP), second to fifth proximal interphalangeal (PIP), first to fifth metacarpophalangeal (MCP), thumb interphalangeal (IP) and thumb base (carpometacarpal joint) joints were graded using a modified Kellgren-Lawrence (KL) scale for hand OA ^18^ as showed in **Supplementary Fig. S11**.

A single musculoskeletal researcher (primary reader, TY, radiologist) read all the hand radiographs. With each new batch of radiographs (n=50), we commingled five previously read radiographs to test intra-rater reliability. To assess the inter-rater reliability of the scoring, another reader (Abasiama D Obotiba, musculoskeletal imaging specialist) scored a selected subset of 30 films independently. Intra- and inter-rater reliabilities were assessed using kappa statistic and 95% CI. Hand OA was defined as the presence of a KL grade≥2 in any of the joints listed above in each hand. The intra-rater and inter-rater reliabilities for hand OA as a dichotomous variable expressed by the kappa statistic were 0.91 (95% CI: 0.83-0.99) and 0.71 (95% CI: 0.45-0.96), respectively.

***Statistical Analysis***

Continuous data was expressed as mean ± SD, and categorical data was expressed as proportion (percentage). Serum ferritin concentrations were classified into five categories according to the quintile distribution: ≤125 μg/L, 125-214 μg/L, 214-302 μg/L, 302-465 μg/L, and >465 μg/L for men, and ≤97 μg/L, 97-147 μg/L, 147-214 μg/L, 214-314 μg/L, and >314 μg/L for women, respectively. The association between serum ferritin levels and hand OA was evaluated by logistic regression model using generalized estimating equations (GEE) ^19^ (hand specific analysis). Both crude ORs and age-sex-BMI adjusted ORs and related 95% CIs of hand OA among different categories of serum ferritin were calculated with the lowest quintiles of serum ferritin category as the reference. The linear trends were tested using the median value of serum ferritin in each category in the logistic regression model. SAS V.9.4 (SAS Institute, Cary, North Carolina, USA) was used. *P* values quoted are 2-sided and *P*<0.05 was considered statistically significant.

1. Wolock, S. L., Lopez, R. & Klein, A. M. Scrublet: Computational Identification of Cell Doublets in Single-Cell Transcriptomic Data. *Cell Syst* **8**, 281-291.e289, doi:10.1016/j.cels.2018.11.005 (2019).

2. Satija, R. *et al.* Spatial reconstruction of single-cell gene expression data. *Nat Biotechnol* **33**, 495-502, doi:10.1038/nbt.3192 (2015).

3. Stuart, T. *et al.* Comprehensive Integration of Single-Cell Data. *Cell* **177**, 1888-1902.e1821, doi:10.1016/j.cell.2019.05.031 (2019).

4. Van der Maaten, L. & Hinton, G. J. J. o. m. l. r. Visualizing data using t-SNE. **9** (2008).

5. Cao, J. *et al.* The single-cell transcriptional landscape of mammalian organogenesis. *Nature* **566**, 496-502, doi:10.1038/s41586-019-0969-x (2019).

6. Emdin, C. A., Khera, A. V. & Kathiresan, S. Mendelian Randomization. *Jama* **318**, 1925-1926, doi:10.1001/jama.2017.17219 (2017).

7. Smith, G. D. & Ebrahim, S. 'Mendelian randomization': can genetic epidemiology contribute to understanding environmental determinants of disease? *Int J Epidemiol* **32**, 1-22, doi:10.1093/ije/dyg070 (2003).

8. Võsa, U. *et al.* Large-scale cis- and trans-eQTL analyses identify thousands of genetic loci and polygenic scores that regulate blood gene expression. *Nat Genet* **53**, 1300-1310, doi:10.1038/s41588-021-00913-z (2021).

9. Sudlow, C. *et al.* UK biobank: an open access resource for identifying the causes of a wide range of complex diseases of middle and old age. *PLoS Med* **12**, e1001779, doi:10.1371/journal.pmed.1001779 (2015).

10. Zhu, Z. *et al.* Integration of summary data from GWAS and eQTL studies predicts complex trait gene targets. *Nat Genet* **48**, 481-487, doi:10.1038/ng.3538 (2016).

11. Funck-Brentano, T. *et al.* Causal Factors for Knee, Hip, and Hand Osteoarthritis: A Mendelian Randomization Study in the UK Biobank. *Arthritis Rheumatol* **71**, 1634-1641, doi:10.1002/art.40928 (2019).

12. Burgess, S. *et al.* Sensitivity Analyses for Robust Causal Inference from Mendelian Randomization Analyses with Multiple Genetic Variants. *Epidemiology* **28**, 30-42, doi:10.1097/ede.0000000000000559 (2017).

13. Verbanck, M., Chen, C. Y., Neale, B. & Do, R. Detection of widespread horizontal pleiotropy in causal relationships inferred from Mendelian randomization between complex traits and diseases. *Nat Genet* **50**, 693-698, doi:10.1038/s41588-018-0099-7 (2018).

14. Bowden, J. *et al.* Improving the accuracy of two-sample summary-data Mendelian randomization: moving beyond the NOME assumption. *Int J Epidemiol* **48**, 728-742, doi:10.1093/ije/dyy258 (2019).

15. Wei, J. *et al.* Association Between Gut Microbiota and Elevated Serum Urate in Two Independent Cohorts. *Arthritis Rheumatol* **74**, 682-691, doi:10.1002/art.42009 (2022).

16. Wei, J. *et al.* Association Between Gut Microbiota and Symptomatic Hand Osteoarthritis: Data From the Xiangya Osteoarthritis Study. *Arthritis Rheumatol* **73**, 1656-1662, doi:10.1002/art.41729 (2021).

17. Zeng, C. *et al.* Dose-response relationship between lower serum magnesium level and higher prevalence of knee chondrocalcinosis. *Arthritis Res Ther* **19**, 236, doi:10.1186/s13075-017-1450-6 (2017).

18. Haugen, I. K. *et al.* Prevalence, incidence and progression of hand osteoarthritis in the general population: the Framingham Osteoarthritis Study. *Ann Rheum Dis* **70**, 1581-1586 (2011).

19. Zhang, Y., Glynn, R. J. & Felson, D. T. Musculoskeletal disease research: should we analyze the joint or the person? *J Rheumatol* **23**, 1130-1134 (1996).

**Supplementary Figures**


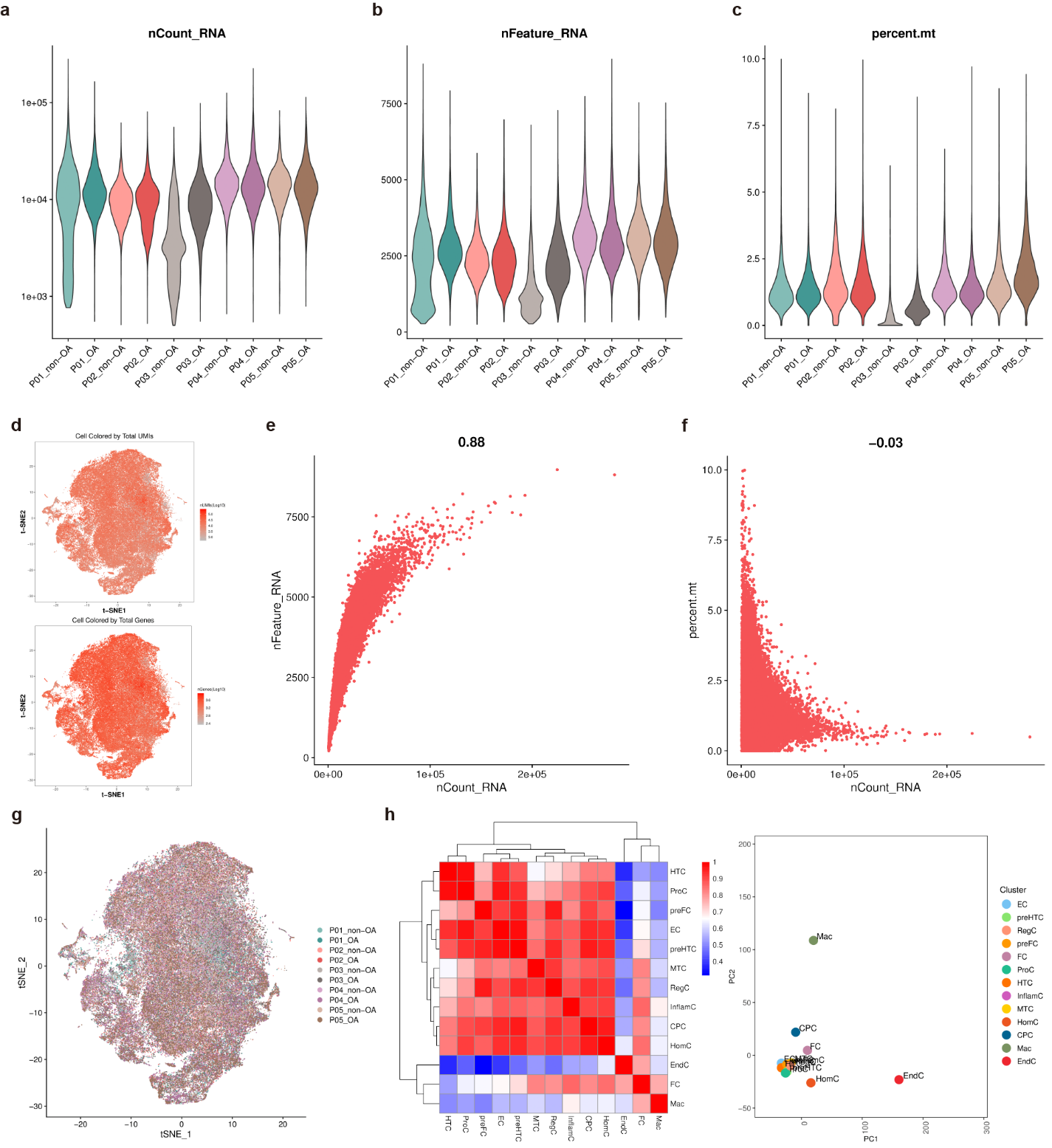


**Supplementary Fig. S1. Quality control for scRNA-seq datasets.**

Violin plots showing the number of RNA counts (a), genes (b), and percent of mitochondrial genes (c) in each sample. (d) tSNE plots showing the number of RNA counts and genes in each cell. (e and f) The correlation between RNA counts and genes, RNA counts and mitochondrial genes in cells. (g) tSNE plots of cells colored according to the samples. (h) The correlation among the annotated subpopulations.


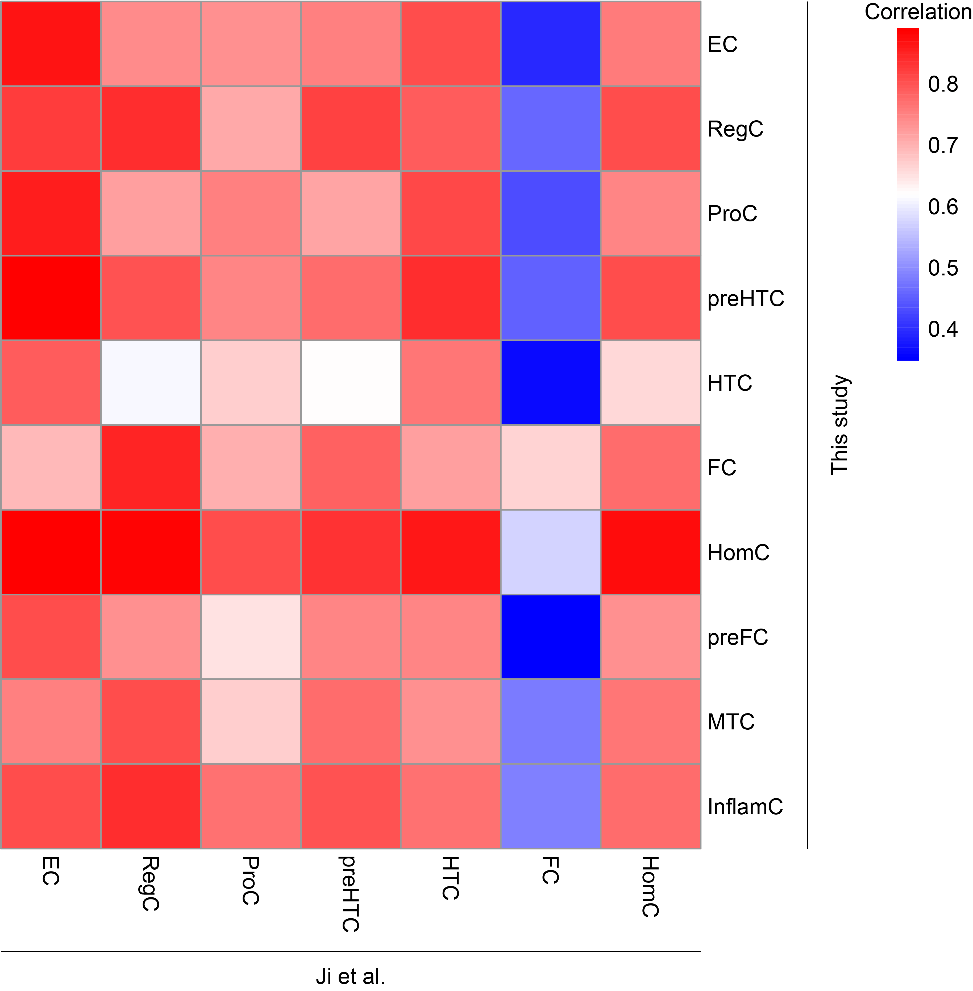


**Supplementary Fig. S2. Pearson pairwise correlations in the global transcriptome between hand and knee articular chondrocytes.** EC, effector chondrocytes; preHTC, prehypertrophic chondrocytes; RegC, regulatory chondrocytes; preFC, prefibrocartilage chondrocytes; FC, fibrocartilage chondrocytes; HTC, hypertrophic chondrocytes; InflamC, inflammatory chondrocytes; MTC, mitochondrial chondrocytes; HomC, homeostatic chondrocytes; CPC, cartilage progenitor cells.


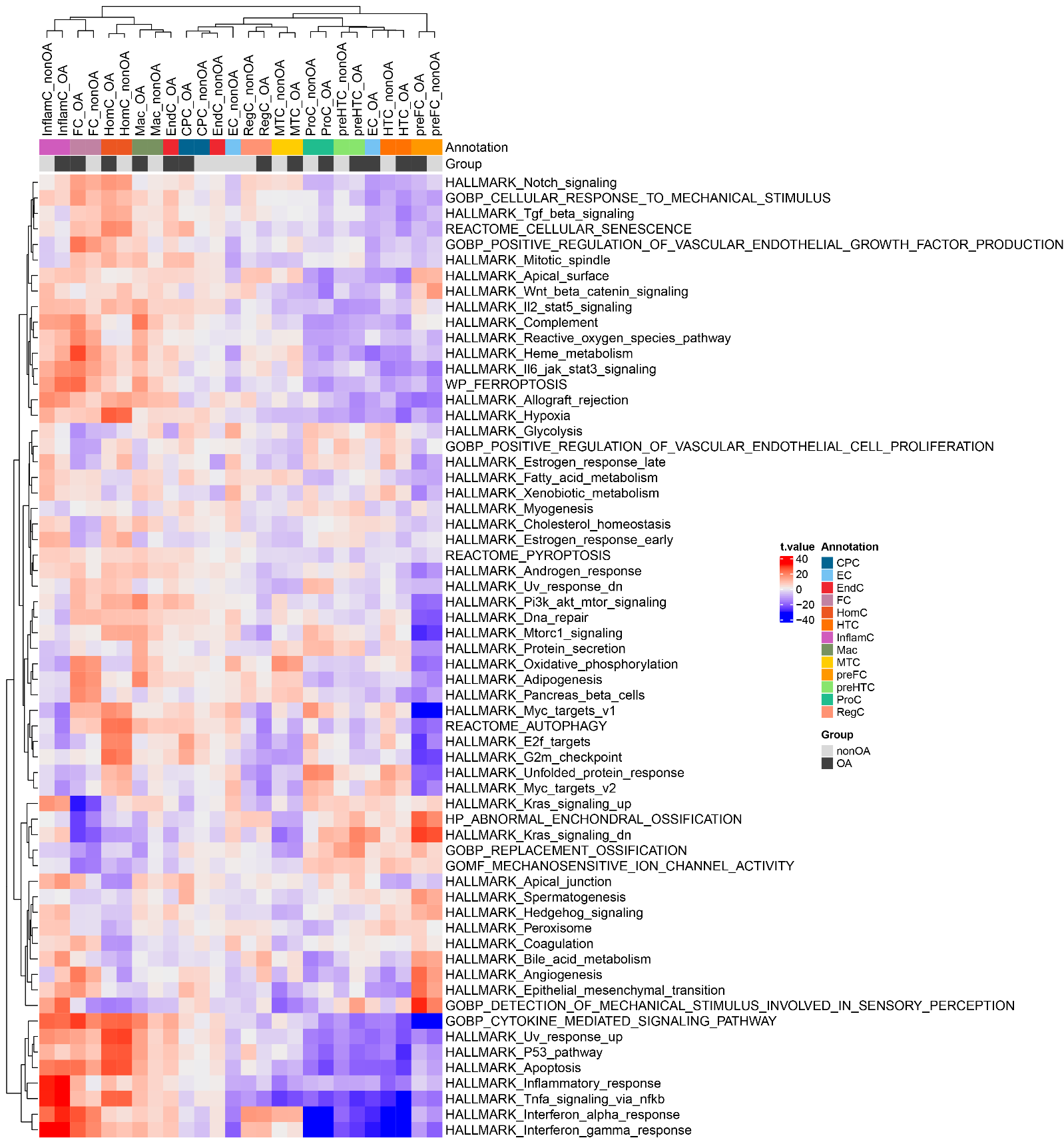


**Supplementary Fig. S3. Gene set variation analysis of cell subpopulations in hand osteoarthritic and non-osteoarthritic cartilage.**


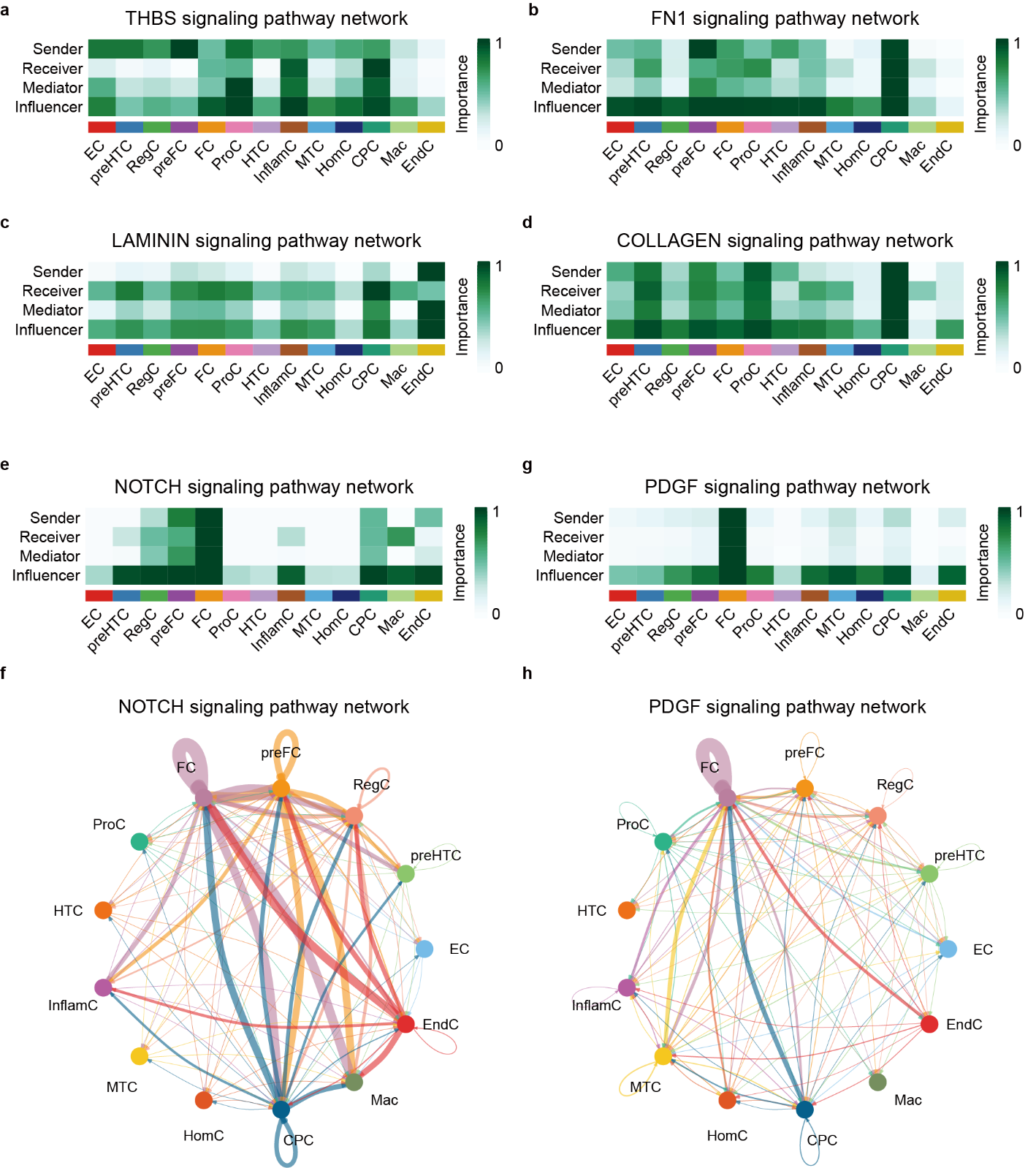


**Supplementary Fig. S4. Cellular communication across cell subpopulations based on ligand-receptor interaction.**

(a-d) Heatmap showing the active participance of chondrocyte subpopulations in the ECM-receptor signalling pathways. Heatmap (e) and circle plot (f) showing a significant role of FC in NOTCH signalling pathway. Heatmap (g) and circle plot (h) showing a significant role of FC in platelet-derived growth factor signalling pathway. EC: effector chondrocytes; preHTC: prehypertrophic chondrocytes; RegC: regulatory chondrocytes; preFC: prefibrocartilage chondrocytes; FC: fibrocartilage chondrocytes; HTC: hypertrophic chondrocytes; InflamC: inflammatory chondrocytes; MTC: mitochondrial chondrocytes; HomC: homeostatic chondrocytes; CPC: cartilage progenitor cells; Mac: macrophages; EndC: endothelial cells.


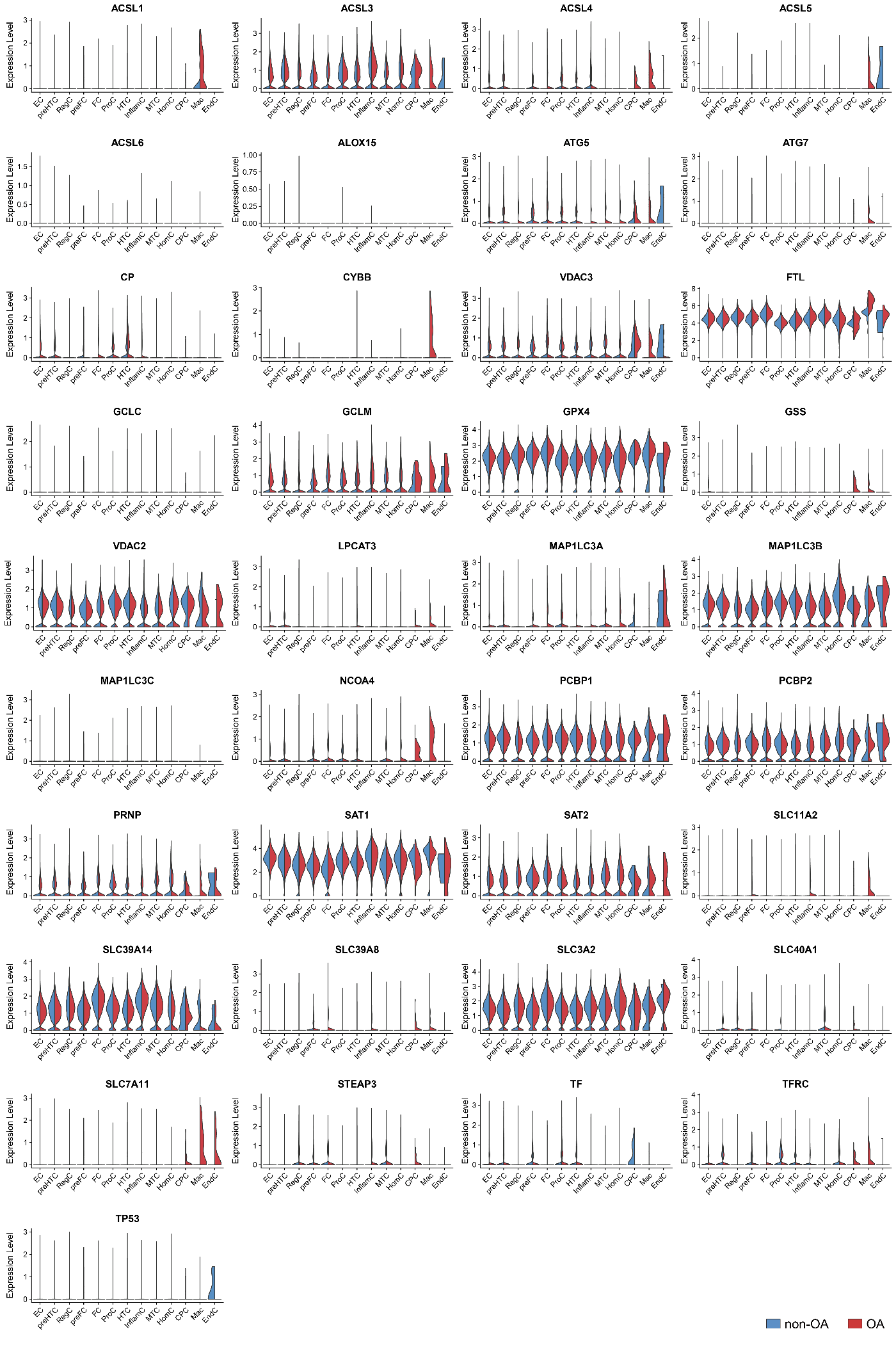


**Supplementary Fig. S5. Differential expression of core ferroptotic genes in all subpopulations between hand osteoarthritic and non-osteoarthritic cartilage.**


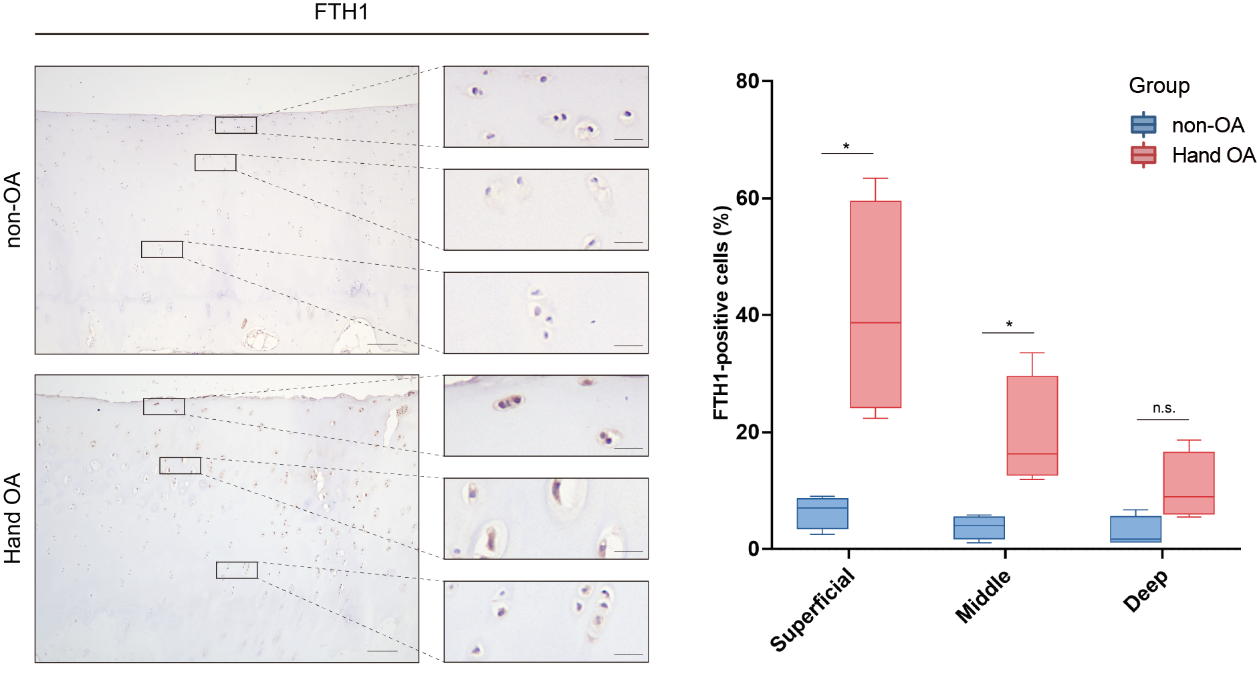


**Supplementary Fig. S6. Representative immunohistochemistry staining for FTH1 between hand OA and non-OA cartilage.** Scale bar, left, 100 μm; right, 20 μm. *P<0.05. OA, osteoarthritis.

**
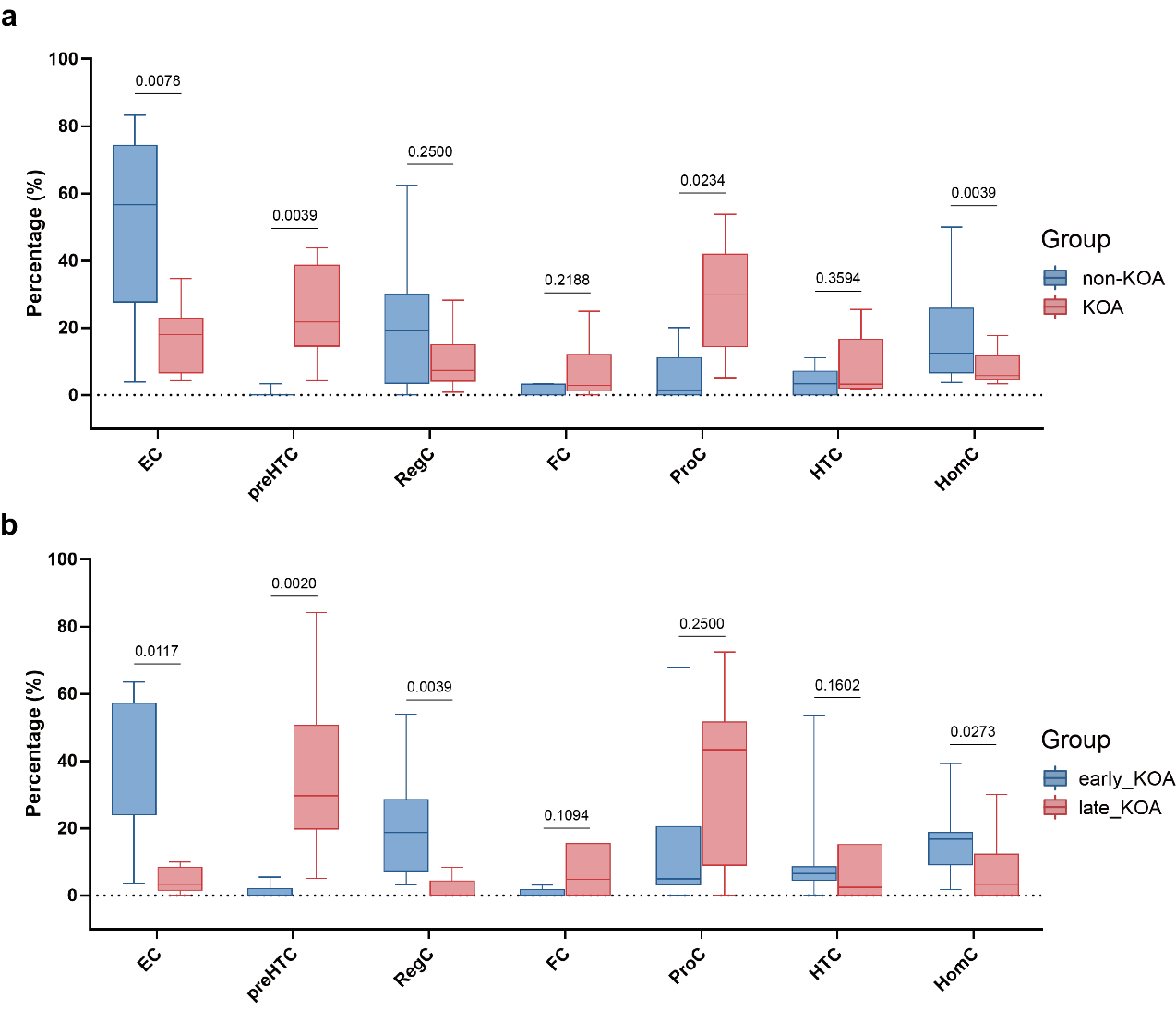
**

**Supplementary Fig. S7. Knee OA-related alterations in subpopulations by scRNA-seq analysis from Ji et al.** (a) Differential analysis of subpopulation abundance between knee OA (stage 1-4) and non-OA cartilage (stage 0). (b) Differential analysis of subpopulation abundance between early knee OA (stage 0-1) and late knee OA (stage 3-4) cartilage. Wilcoxon matched-pairs signed rank test was used for analysis, and a P value<0.05 is considered significant. KOA, knee osteoarthritis; EC, effector chondrocytes; preHTC, prehypertrophic chondrocytes; RegC, regulatory chondrocytes; FC, fibrocartilage chondrocytes; HTC, hypertrophic chondrocytes; HomC, homeostatic chondrocytes.

**
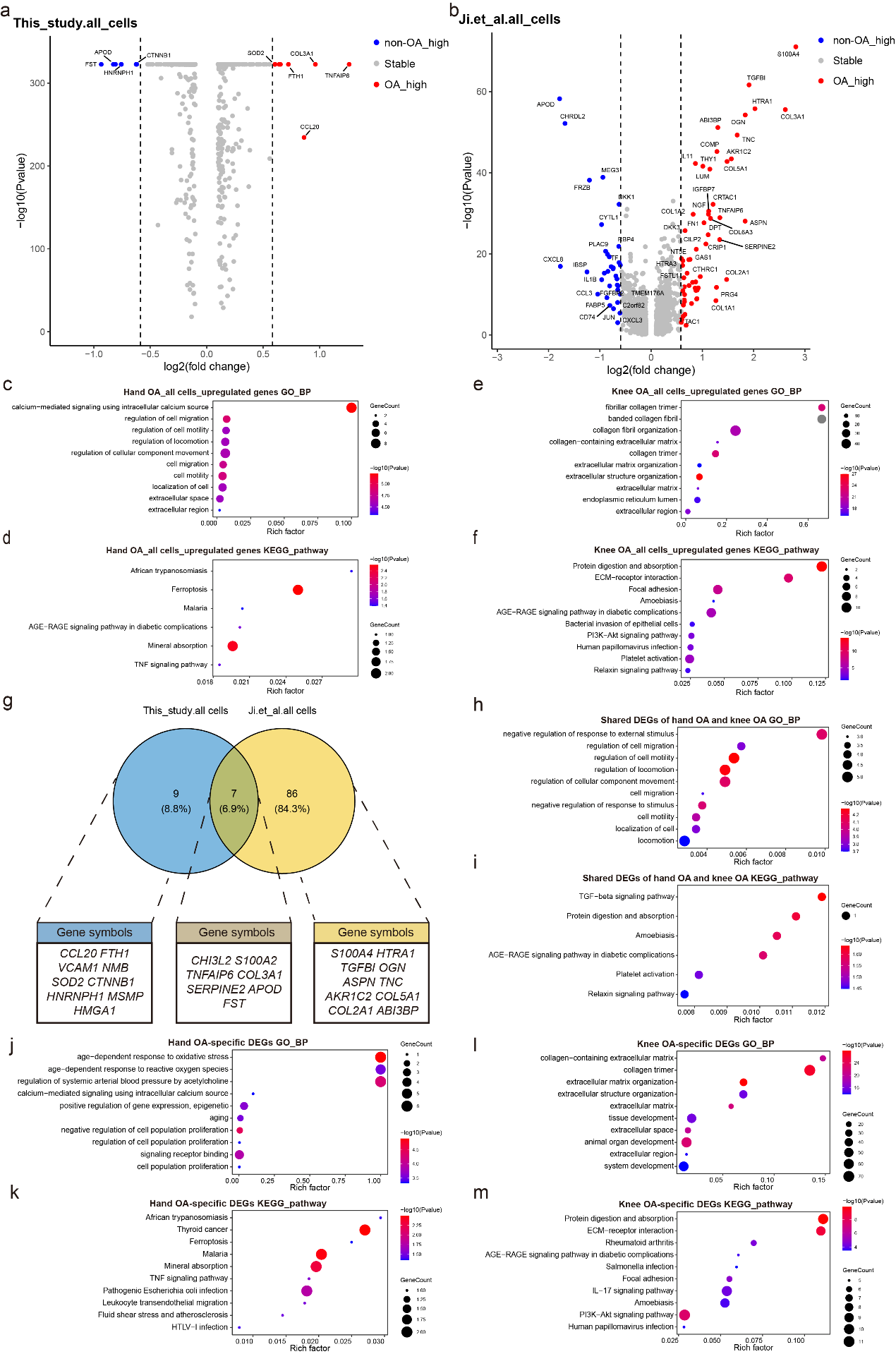
**

**Supplementary Fig. S8. Differences in molecular alterations of all cells between hand OA and knee OA.** (a) Volcano plot showing DEGs of all cells between OA and non-OA cartilage of the hand (|log fold change|>0.5; P<0.05). (b) Volcano plot showing DEGs of all cells between OA and non-OA cartilage of the knee (Ji et al.,). (c and d) GO and KEGG enrichment analysis of the upregulated genes in hand OA. (e and f) GO and KEGG enrichment analysis of the upregulated genes in knee OA. (g) Venn plot showing the similarities and differences in OA-related genes between hand OA and knee OA cartilage (top 10 DEGs are listed in each category). (h and i) GO and KEGG enrichment analysis of the shared DEGs of hand OA and knee OA. (j and k) GO and KEGG enrichment analysis of hand OA-specific DEGs. (l and m) GO and KEGG enrichment analysis of knee OA-specific DEGs. OA, osteoarthritis; DEGs, differentially expressed genes; GO, gene ontology; KEGG, Kyoto Encyclopedia of Genes and Genomes.

**
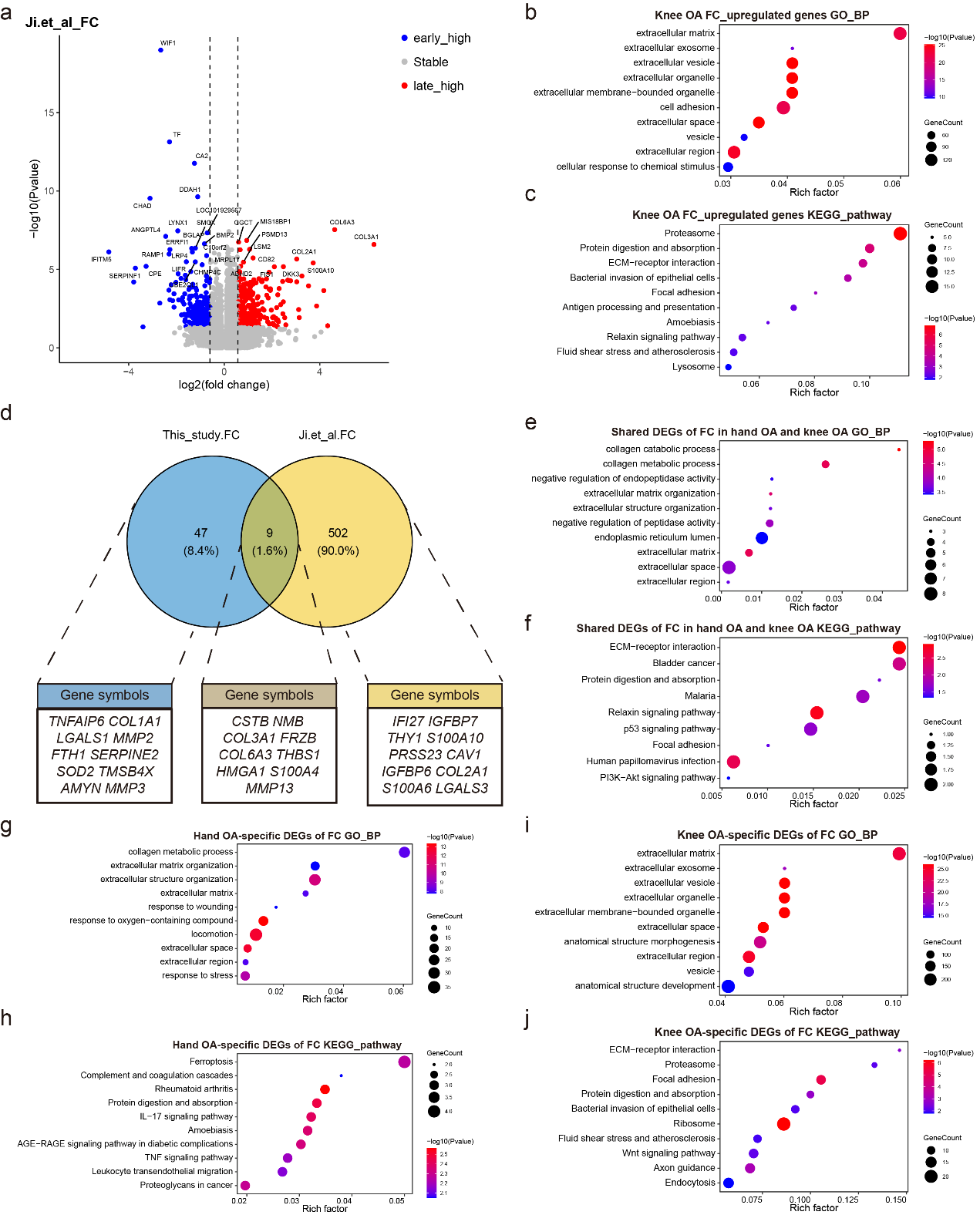
**

**Supplementary Fig. S9. Differences in molecular alterations of FC between hand OA and knee OA.** (a) Volcano plot showing DEGs of FC between early-stage and late-stage OA cartilage of the knee (Ji et al.,) (|log fold change|>0.5; P<0.05). (b and c) GO and KEGG enrichment analysis of the upregulated genes of FC in knee OA. (d) Venn plot showing the similarities and differences in OA-related genes between hand OA and knee OA cartilage. (e and f) GO and KEGG enrichment analysis of the shared DEGs of FC in hand OA and knee OA (top 10 DEGs are listed in each category). (g and h) GO and KEGG enrichment analysis of hand OA-specific DEGs of FC. (i and j) GO and KEGG enrichment analysis of knee OA-specific DEGs of FC. OA, osteoarthritis; DEGs, differentially expressed genes; FC, fibrocartilage chondrocytes; GO, gene ontology; KEGG, Kyoto Encyclopedia of Genes and Genomes.


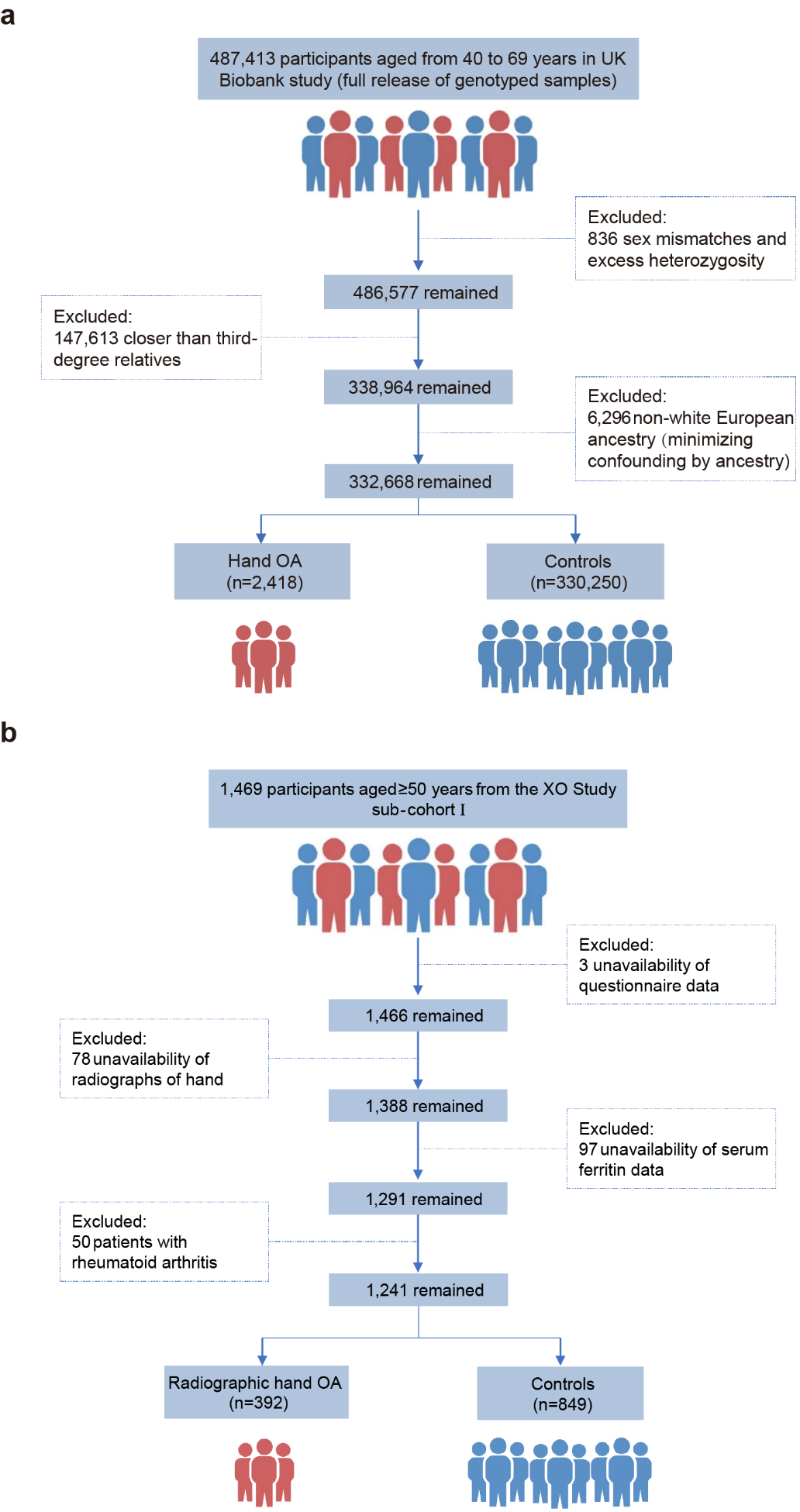


**Supplementary Fig. S10. Selection process of included subjects for the population-based studies.**

(a) Selection process of included subjects for the MR analysis in UK Biobank. (b) Selection process of included subjects for the cross-sectional analysis in Xiangya OA Study. MR: Mendelian randomisation; OA: osteoarthritis.


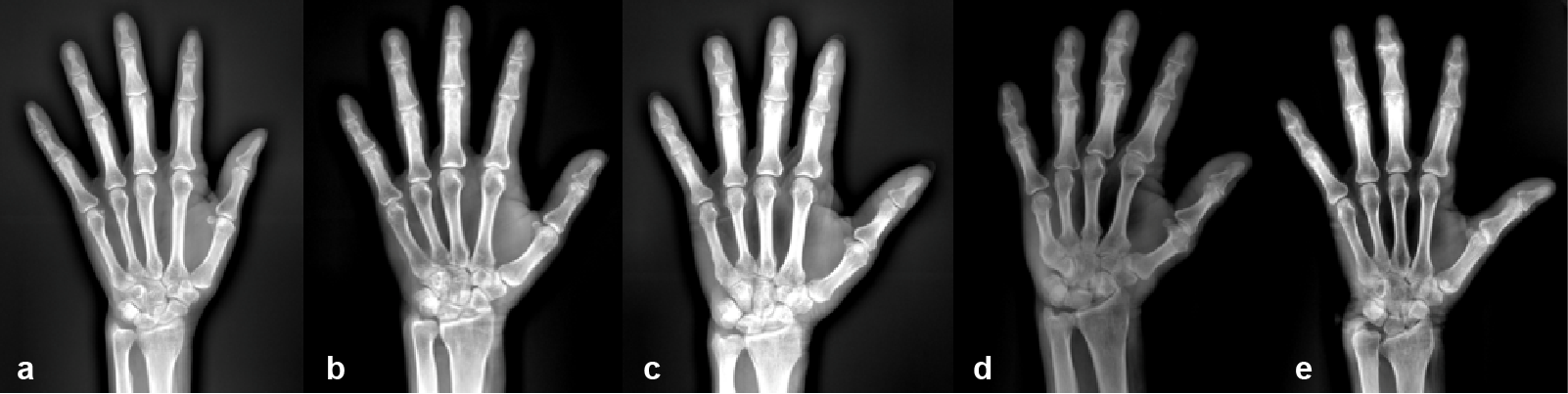


**Supplementary Fig. S11. Modified KL scale for hand OA assessment.**

(a) KL=0: no osteophyte (OP) and/or joint space narrowing (JSN). (b) KL=1: questionable OP(s) and/or JSN. (v) KL=2: small OP(s) and/or mild JSN, sclerosis may be present. (d) KL=3: moderate OP(s) and/or moderate JSN, sclerosis and erosions may be present. (e) KL=4: large OP(s) and/or severe JSN, sclerosis and erosions may be present.
